# A γ-polyglutamic acid-based artificial biofilm for field delivery of Bacillus amyloliquefaciens KNU-28: an uncontrolled multi-orchard demonstration on peach gummosis with exploratory bark fungal community profiling

**DOI:** 10.3389/fpls.2026.1933566

**Published:** 2026-08-31

**Authors:** Min-Kyu Park, Yeong-Jun Park, Tae-Ho Kim, Yujin Hyun, Sook-Min Kwon, Muhyeok Kwon, Tae-Kyung Hwang, Jae-Ho Shin

**Affiliations:** 1 Department of Applied Biosciences, Kyungpook National University, Daegu, Republic of Korea; 2 Kyungpook National University Next-Generation Sequencing (KNU NGS) Core Facility, Kyungpook National University, Daegu, Republic of Korea; 3 Microbalance Inc., Daegu, Republic of Korea; 4 Gunwi Agricultural Technology Center, Gunwi-gun, Daegu, Republic of Korea

**Keywords:** artificial biofilm, Bacillus amyloliquefaciens, biocontrol, Botryosphaeria, fungal microbiome, ITS amplicon sequencing, peach gummosis, γ-polyglutamic acid

## Abstract

Peach gummosis, caused primarily by *Botryosphaeria dothidea sensu lato*, is a destructive canker disease with few environmentally friendly management options. Here we report an uncontrolled pre/post field demonstration of a γ-polyglutamic acid-based artificial biofilm — a biodegradable delivery platform whose agent-agnostic design remains untested — encapsulating *Bacillus amyloliquefaciens* KNU-28 across 143 trees in 18 commercial peach orchards in South Korea. Severity declined from a pre-treatment mean of 4.12 (April) to 2.32 by August (76.2% of 143 trees improved by ≥1 point) and to 1.78 by October in three regions (88.3% of 128 trees). In an ordinal cumulative-link mixed model, severity fell below the April baseline at every later visit; the average monthly odds ratio was 0.70 (95% CI 0.66–0.75); alternative model specifications agreed. Operator-independent image-based ΔE quantification across 74 trees showed a concordant cohort-level decline (lesion-area declines of 19–30%; significant in one of four regions after FDR correction) but agreed only weakly with the ordinal score at tree level (Spearman ρ = 0.16), and is complementary rather than confirmatory. We sequenced fungal ITS2 from 23 bark samples. Multivariate dispersion differed among bark groups (PERMDISP F = 20.7, p < 0.001), and healthy and gummosis-affected bark did not separate under compositionally aware metrics (Aitchison/robust CLR, p > 0.34); the nominally significant Bray–Curtis PERMANOVA (R^2^ = 0.248, p = 0.001) therefore cannot be read as a centroid difference. Early- and later-treatment diseased communities did not differ (p = 0.542; all genera FDR q > 0.5). Gummosis-enrolled bark showed higher relative abundances of *Wickerhamomyces*, *Cytospora* and *Botryosphaeria* (dominant ASVs assigned to *B. qingyuanensis*) than healthy bark, though none survived FDR correction. Because the 19 diseased libraries came from only 10 trees, the genus–severity correlations were replaced by a paired analysis, in which no genus differed nominally. Together, this demonstration establishes the operational feasibility of field-scale application and a parameter-transparent lesion-quantification method; the severity decline and mycobiota trends are accompanying observations. Because untreated controls, strain-level KNU-28 tracking, verification of delivery to bark and a powered microbiome cohort were absent, the data do not establish causal efficacy and warrant randomized, untreated-control trials.

## 1 Introduction

Gummosis is one of the most economically significant diseases affecting stone fruit orchards worldwide, with affected species including peach (*Prunus persica*), plum (*Prunus salicina*), and cherry (*Prunus avium*). The disease is primarily caused by *Botryosphaeria dothidea* and related Botryosphaeriaceae species, which infect bark tissue, causing the characteristic gum exudation, as well as perennial canker formation and branch dieback (Weaver, 1974; Wang et al., 2011; Phillips et al., 2013). In severe cases, repeated infection leads to tree death within several seasons, resulting in substantial yield and economic losses (Slippers and Wingfield, 2007; Slippers et al., 2007; Wang et al., 2011). Because the pathogen colonizes bark and woody tissue and can persist as latent, endophytic infections, conventional fungicide sprays and pruning often achieve only partial and short-lived control, and the bark-residing nature of *Bo. dothidea* has motivated interest in management strategies that engage the bark microbial community itself (Slippers et al., 2007; Wang et al., 2011). Peach bark canker communities can be polymicrobial; fungi of the Valsaceae (including *Cytospora*) have been detected alongside Botryosphaeriaceae in peach bark (Jo et al., 2022). The species designation *Botryosphaeria dothidea* is used here in the historical broad sense, inferred from characteristic gummosis symptoms and historical isolations from these orchards; molecular confirmation via multilocus phylogeny (ITS, tef1, tub2) was not performed in the current study, and the designation should be understood as *Bo. dothidea sensu lato* (Slippers and Wingfield, 2007; Phillips et al., 2013).

Current management of peach gummosis relies heavily on synthetic fungicides, including tebuconazole and propiconazole, and sanitation practices, such as the pruning of infected material (Slippers et al., 2007). However, increasing public and regulatory concern over pesticide residues in food, soil contamination, and the development of fungicide-resistant pathogen populations has created an urgent demand for biological control alternatives (Janisiewicz and Korsten, 2002; Droby et al., 2009; Compant et al., 2010; Nunes, 2012; Massart et al., 2015; Köhl et al., 2019). Plant growth-promoting rhizobacteria (PGPR) and bacteria of the genus *Bacillus* have emerged as promising biocontrol candidates owing to their production of diverse antifungal metabolites, including iturin, fengycin, bacillomycin, and surfactin lipopeptides (Romero et al., 2007; Ongena and Jacques, 2008; Pérez-García et al., 2011).

**Figure 1:**
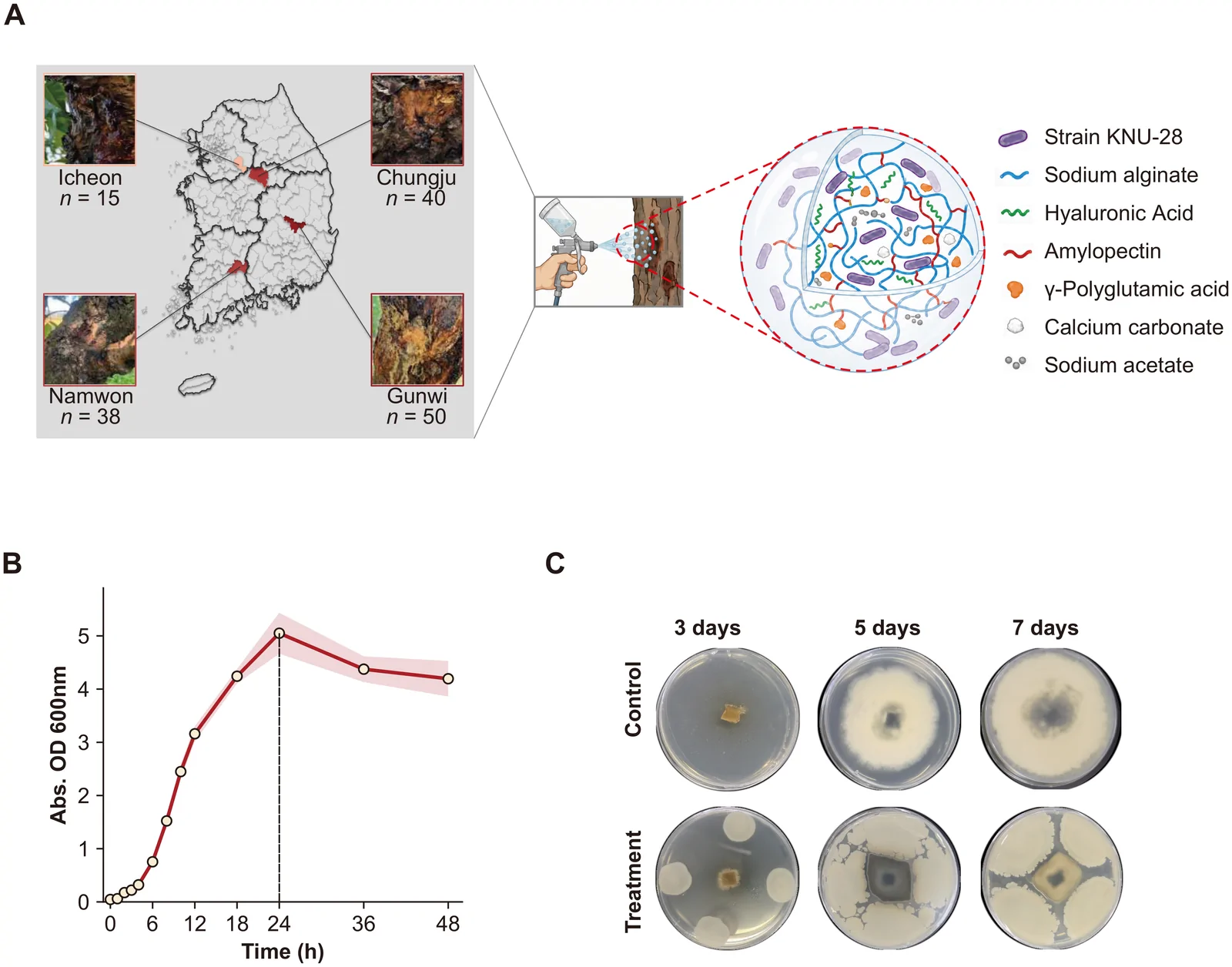
Study overview, artificial biofilm structure, and characterization of *Bacillus amyloliquefaciens* KNU-28. **(A)** Study sites across four administrative regions of South Korea, with a representative active gum-exudate photograph for each region (left), and a schematic cross-section of the Ooho-like artificial biofilm bead showing a γ-PGA/alginate matrix carrying embedded KNU-28 cells and local release on the peach bark lesion surface, with the formulation components listed beneath (right). **(B)** Growth curve of *Ba. amyloliquefaciens* KNU-28 in Luria–Bertani broth at 30 °C, 200 rpm. Data points represent the mean ± SD of three independent replicates, with the dashed vertical line indicating peak OD600 value (~5.05) reached at 24 h. **(C)** Antifungal activity against *Botryosphaeria dothidea*, assessed in dual-culture assays on PDA at 3, 5, and 7 days post-inoculation. The upper-row images show *Bo. dothidea* alone (control), and the lower-row images show *Bo. dothidea* in co-cultures with KNU-28 (treatment). Inhibition zones are visible starting at day 5.

*Bacillus amyloliquefaciens*, with demonstrated activity against a broad spectrum of plant pathogens, is among the most extensively studied biocontrol species (Ongena and Jacques, 2008; Li et al., 2016). Strain KNU-28 was isolated from the leaves of a peach tree (*Prunus persica*) in Apo-eup, Gimcheon-si, Gyeongsangbuk-do, Republic of Korea (Kim et al., 2022). The strain was originally deposited as *Bacillus amyloliquefaciens*, and the species assignment follows the original genome-announcement record (Fan et al., 2017; Kim et al., 2022). It exhibited strong antifungal activity in dual-culture assays (**Figure 1C**) against a *Botryosphaeria dothidea* reference isolate obtained from the Korean Agricultural Culture Collection (KACC45481) and was previously identified as a candidate biocontrol agent for gummosis management (Kim et al., 2022). A key limitation of conventional biocontrol formulations, however, is their poor persistence on plant surfaces under field conditions, which reduces the duration of protection (Köhl et al., 2019; Saberi Riseh et al., 2021). Encapsulation technologies that prolong cell viability and maintain close contact with target surfaces can substantially improve field efficacy (Saberi Riseh et al., 2021). Recent work has extended this approach with multilayer biopolymer capsules, protective hydrogels and chitosan gel beads that keep plant-beneficial and biocontrol bacteria viable through months of storage and improve their establishment and performance in planta (Feng et al., 2025; Krastev et al., 2025; Buttrós et al., 2026; Saberi Riseh and Fathi, 2026). Those systems have, however, been developed almost exclusively for seed, root or soil delivery; carriers designed to adhere to and persist on woody bark surfaces remain comparatively unexplored.

We developed an Ooho-like artificial biofilm formulation in which KNU-28 cells are embedded in a biodegradable matrix composed of γ-polyglutamic acid (γ-PGA), alginate, hyaluronic acid, calcium carbonate, sodium acetate, and amylopectin (Liu et al., 2010; Yu et al., 2016). The biofilm is designed to adhere to bark surfaces, provide a sustained release of biocontrol agents, and degrade without environmental persistence. The formulation has been patented (Korean Patent Application No. 10-2023-0051762).

Despite growing interest in microbiome-mediated biocontrol mechanisms, relatively few studies have characterized how biocontrol agent applications alter the resident fungal community on plant bark. The bark microbiome is an important but understudied component of the plant phytobiome, and biocontrol agent-associated shifts in phytobiome composition can reflect both direct antagonism by the agent and broader ecological changes associated with reduced disease pressure (Ren et al., 2019; Jo et al., 2022). Jo et al. (2022) reported differences in bark fungal community structure between healthy and gummosis-affected peach trees in Korea. The ITS component of the present study does not supersede that cross-sectional description: it extends it only by adding a treatment time series, characterizing diseased bark mycobiota at two timepoints during a field application rather than at a single point, and it is accordingly positioned as exploratory baseline characterization of the treated diseased bark mycobiota — a prerequisite for interpreting any future treatment-associated change — rather than as a novel description of the gummosis mycobiome. To our knowledge, however, no previous study has applied a γ-PGA biofilm-encapsulated *Bacillus* biocontrol agent at field scale across multiple commercial orchards while profiling the bark mycobiota across the treatment season. Addressing this gap, we conducted multi-site treatments across 18 commercial orchards in four administrative regions of South Korea, combining monthly disease-severity monitoring (April–October 2025) with bark ITS amplicon sequencing, to (i) characterize the field response of peach gummosis to an artificial biofilm-based KNU-28 treatment and (ii) describe the bark fungal community of peach gummosis at field scale relative to healthy bark and explore early- and later-treatment compositional trends as a hypothesis-generating analysis in an exploratory microbiome subset (n = 23 libraries), and (iii) report an operator-independent, parameter-transparent image-based measure of bark lesion area and quantify its agreement with the field severity score. This study is framed as an uncontrolled, multi-orchard pre/post demonstration trial — without concurrent untreated control trees — designed to generate field-scale hypotheses for subsequent randomized, controlled efficacy testing.

## 2 Materials and methods

### 2.1 Bacterial strain and artificial biofilm preparation

*Bacillus amyloliquefaciens* KNU-28 was cultured in Luria–Bertani broth at 30 °C with shaking at 200 rpm. Growth kinetics measurement confirmed that peak optical density (OD_600_ ≈ 5.05) was achieved at 24 h of incubation (**Figure 1B**), after which cultures were harvested for biofilm-based biocontrol agent formulation.

The artificial biofilm was prepared as an Ooho-like biodegradable encapsulation formulation. The matrix comprised the following components (concentrations expressed as final w/v in the composite matrix): γ-polyglutamic acid (γ-PGA; 2.5–5% w/v), which functions as the primary matrix polymer and biofilm-promoting scaffold for *Bacillus* cells (Liu et al., 2010; Ogunleye et al., 2015; Yu et al., 2016); sodium alginate (1% w/v), which provides the structural gel backbone cross-linkable by divalent cations (Bashan, 1986); hyaluronic acid (0.1% w/v), which augments matrix viscosity and promotes adhesion to bark surfaces; calcium carbonate (0.5% w/v), which supplies supplementary Ca^2+^ and buffers the matrix pH during gelation; sodium acetate (0.3% w/v), which stabilizes the matrix pH; and amylopectin (1% w/v), which acts as a rheological modifier to adjust bead consistency. The carrier medium was 1/10-strength Luria–Bertani (LB) broth, which provides a minimal nutrient base compatible with bacterial viability during encapsulation. *Bacillus amyloliquefaciens* KNU-28 cells, harvested at peak OD_600_ at 24 h of incubation, were incorporated into the composite matrix before gelation. Ooho-type bead gelation was initiated by adding a 2% (w/v) CaCl_2_ solution at 10% (v/v) of the composite matrix volume; the Ca^2+^ ions cross-link the alginate chains to form a semi-solid, spherical bead structure encapsulating the bacterial cells (**Figure 1A**). The compatibility of KNU-28 with the γ-PGA matrix was qualitatively checked by an *in vitro* concentration screen on 1/10-strength LB agar (**Supplementary Figure 3**; see the Methods below), which indicated that the 2.5–5% (w/v) γ-PGA range was compatible with visible colony formation without measurable pH perturbation (ΔpH ≈ 0.12 across the full 2.5–15% w/v range tested). Component ratios and gelation conditions beyond those stated above — including the precise sequence and timing of component addition — constitute the patent-protected core of the formulation and are disclosed in the embodiment examples of Korean Patent Application Nos. 10-2025-0181627 and 10-2024-0155465; academic non-commercial access for confirmatory studies can be requested through the patent holder, Microbalance Inc. (Daegu, Republic of Korea), under a material transfer agreement addressed to the corresponding author. For each treatment application, freshly prepared Ooho-like beads were applied by direct spraying onto gummosis-affected bark wound surfaces at a delivered dose of approximately 5 × 10^5^ CFU cm^-2^ of bark canopy (estimated from the OD_600_ 5.05 peak culture titer of approximately 2 × 10^9^ CFU mL^–1^ diluted 1:10^3^ in the bead suspension, applied at 30–50 mL per 150–200 cm^2^ bark area; this estimate was not independently verified by direct CFU enumeration of the beads). Three assumptions constrain that figure and should be read with it: the OD_600_-to-CFU conversion is a generic factor that was not calibrated for KNU-28; the per-tree spray volume (30–50 mL) and the bark area (150–200 cm^2^) are a target volume and an estimated area rather than measured quantities; and encapsulation can reduce the number of viable cells actually delivered, potentially substantially. The figure should therefore be read as an order-of-magnitude upper-bound estimate of the delivered dose rather than as a measured titer. Treatments were administered once at the April baseline visit and then re-applied once monthly on each subsequent visit through October, with a per-tree spray volume of 30–50 mL targeted at the visibly affected bark area; spray equipment consisted of hand-held compressed-air sprayers operated by the same two assessors who recorded severity. The beads were soft and freshly gelled and were delivered as a suspension rather than as rigid spheres, which is what makes spray application possible.

Prior to formulation lock-in, the γ-PGA matrix concentration was screened *in vitro* on a quarter-log range of 2.5%, 5%, 10%, and 15% (w/v) in 1/10-strength LB supplemented with 1.5% (w/v) agar (150 mL per condition; autoclaved at 121°C for 15 min before plating). For each plate, the medium pH was measured immediately after pouring, and *Ba. amyloliquefaciens* KNU-28 from a single LB-agar colony was zigzag-streaked across the surface and incubated at 30°C for 22 h. Several parameters of this screen were neither optimised nor characterised: the 22 h read time was not calibrated against a growth curve on γ-PGA plates; colony density was scored qualitatively rather than by counting or by zone-of-inhibition measurement; medium pH was not re-measured at the end of incubation; the molecular weight of γ-PGA after autoclaving was not determined; and growth on an agar surface does not reproduce the microenvironment experienced by cells inside a bead. The screen therefore establishes matrix compatibility only, and not intra-bead viability. Plate-level pH readings and qualitative colony density on streaked plates were used to evaluate whether γ-PGA concentration perturbed medium pH and KNU-28 viability across the candidate matrix range. This screen was a single-batch pilot (n = 1 per concentration) and is reported as **Supplementary Figure 3**; concentrations that supported visibly robust colony formation in this qualitative single-batch screen (the primary compatibility criterion; medium pH remained near-neutral across the full tested range, 6.81–6.93, with no discriminating pH signal) were retained as the preliminary working range for the Ooho-form formulation described above.

### 2.2 Field trial design

Field trials were conducted in four administrative regions of South Korea duringApril–October 2025. The study sites included orchards in Gunwi (Daegu; n = 50 trees), Namwon (Jeonbuk-do; n = 38), Chungju (Chungcheongbuk-do; n = 40), and Icheon (Gyeonggi-do; n = 15), for a pooled sample size of n = 143 trees across 18 farms (**Supplementary Table 1**). All experimental trees were peach trees (*Prunus persica*) of the yellow-fleshed cultivar ‘Hwangdo’, the only cultivar planted across the 18 participating orchards, and were selected on the presence of confirmed gummosis lesions in April; their April baseline severity ranged from 2 to 5, with 108 of the 143 trees (75.5%) scoring 4 or 5. For disease severity assessments, no untreated control plots or trees were used; instead, pre-treatment April measurements served as the within-tree baseline. This design is, thus, a pre/post demonstration trial without concurrent no-treatment control group (see Limitations in Discussion). No randomisation was performed at any level: trees were enrolled on the basis of the April severity criterion rather than allocated, and every enrolled tree received the treatment. Throughout the observation period the enrolled trees also remained under the participating growers’ routine orchard management, including any conventional spray programmes, irrigation and pruning applied at each site; these programmes were not recorded for this study.

Biofilm treatments were first applied at the April baseline visit and subsequently administered once monthly through October at all four sites. Monthly assessments were conducted from April to October 2025 at the Gunwi, Namwon, and Chungju sites. At the Icheon site, clinical severity scoring was conducted from April to August 2025 and was discontinued thereafter; monthly photographs for the image analysis continued through October, so Icheon contributes seven monthly tiles per tree to the image cohort but no September or October severity scores. Disease severity was assessed at each visit by two trained assessors (T-HK and M-KP), who also applied the treatment and were blinded to the prior month’s score (all enrolled trees were treated, so blinding to treatment status was not applicable), using a five-stage scale based on lesion diameter and per-tree lesion count. Scores were recorded as consensus field scores, and formal inter-rater reliability statistics were not calculated:

- Score 1: No visible lesions (complete recovery).
- Score 2: Lesion diameter < 1 cm; lesion count < 3.
- Score 3: Lesion diameter 1–3 cm; lesion count 3–5.
- Score 4: Lesion diameter 3–5 cm; lesion count 5–10.
- Score 5: Lesion diameter > 5 cm; lesion count > 10.

For each tree, improvement was recorded when a post-baseline severity score was lower than its own April baseline score. The regional improvement rate was defined as the proportion of trees showing improvement at the given assessment month. Overall within-tree changes in severity scores across the treatment period, combining measurements from all trees, were statistically assessed for April vs. August in the full four-region cohort and April vs. October in the three regions assessed through the end of the growing season using a paired Wilcoxon signed-rank test. Mean per-tree severity score changes in a given month were compared between regions using Kruskal–Wallis tests, with Benjamini–Hochberg correction across the monthly contrasts; the corrected regional contrasts are not reported in the Results because no regional difference is claimed. The within-tree paired Wilcoxon contrasts of each month against the April baseline were likewise Benjamini–Hochberg corrected across the six monthly comparisons. Because severity is an ordinal score, the primary model was an ordinal cumulative-link mixed model with nested random intercepts (CLMM; R package ordinal, function clmm), which makes no normality assumption about residuals and represents the repeated monthly observations as nested within trees and trees as nested within orchards (143 trees in 18 farms). Because a month-as-factor specification fitted substantially better than a linear time term (Section 3.1), the primary model treats month as a categorical fixed effect with April as the reference level [severity ~ factor(month) + (1 | farm) + (1 | tree)]; a linear-month CLMM [severity ~ months_since_baseline + (1 | farm) + (1 | tree)] is retained only as a secondary summary of the average seasonal rate, and its time effect is reported on the log-odds scale together with the corresponding odds ratio per month. The contribution of the tree-level random intercept was assessed by a likelihood-ratio test of the nested model against the farm-only specification. Simpler specifications were retained only as sensitivity analyses: single-random-effect CLMMs carrying either the farm-level or the tree-level random intercept alone, a linear mixed-effects model (severity ~ months_since_baseline + (1 | farm); ML estimation in Python statsmodels v0.14), a generalized estimating equation (GEE) model with exchangeable working correlation and cluster-robust standard errors clustered at the farm level, and — to represent the longitudinal structure explicitly rather than assuming equal correlation between all timepoints — a generalized least-squares model with a first-order autoregressive within-tree correlation (AR(1)); the ordinal and autoregressive models were fitted in R (packages ordinal and nlme), following the guidance of Bolker et al. (2009) for ecological repeated-measures data.

### 2.3 Fungal microbiome analysis

For bark fungal community profiling, ITS amplicon sampling was carried out at 11 of the 18orchards (one to five per administrative region): diseased-tree libraries came from eight orchards (GW_D, GW_E, GW_F, NW_B, CJ_D, CJ_E, IC_A and IC_C) and healthy-bark libraries from four orchards (GW_A, GW_C, CJ_A and IC_A), one of which (IC_A) contributed both (**Supplementary Table 1**). Bark samples were collected from healthy control peach trees in May (Healthy; n = 4), fromgummosis-affected peach trees at an early treatment timepoint in May (May; n = 9), and fromgummosis-affected peach trees at a later treatment timepoint in July (July; n = 10). Because biofilm treatment was first applied at the April baseline visit (see Methods), both diseased sampling points postdate treatment initiation; the May and July groups therefore represent early (approximately 1 month) versus later (approximately 3 months) treatment rather than a pre- versus post-treatment contrast, and no untreated diseased baseline mycobiota was sampled. This study therefore relies on two distinct, non-interchangeable reference points—the within-tree April baseline used for the clinical severity analysis (see Methods; a pre/post reference rather than an untreated control) and the healthy-tree group (Healthy) used as a case-control comparator for the bark mycobiota—and neither constitutes a concurrent untreated control. The outermost woody tissue was removed and an approximately 1 cm-diameter section of the exposed bark surface was scraped to collect tissue. Bark for the May round was collected on 28 April 2025, two to three days earlier than the planned 1 May visit, and bark for the July round on 25 July 2025; the two groups are named May and July after the monthly assessment rounds in which they were taken. Total genomic DNA was extracted using the DNeasy PowerSoil Pro Kit (Qiagen, Hilden, Germany). The ITS2 region was amplified using primers ITS86F (5’-ACACTCTTTCCCTACACGACGCTCTTCCGATCTGTGAATCATCGAATCTTTGAA-3’) and ITS4 (5’-GTGACTGGAGTTCAGACGTGTGCTCTTCCGATCTCCTCCGCTTATTGATATGC-3’) (White et al., 1990; Op De Beeck et al., 2014) and sequenced on an Illumina MiSeq platform as single-end reads of 301 bp, matching the layout and read length recorded for all 23 deposited runs (**Supplementary Tables 3, 6**). Of 30 bark samples submitted for sequencing, 7 (samples 1, 4, 15, 20, 23, 26 and 28) returned no sequence data — no FASTQ files were returned for these samples — and were excluded; the 23 libraries analysed here are therefore the complete set of libraries obtained. Note: each printed primer sequence includes a 5’ Illumina TruSeq adapter overhang prepended to the core functional primer for direct amplicon library preparation; core ITS86F: 5’-GTGAATCATCGAATCTTTGAA-3’; core ITS4: 5’-TCCTCCGCTTATTGATATGC-3’. Raw reads were processed in QIIME 2 (v2025.7.0) using DADA2 for denoising and chimera removal (Callahan et al., 2016; Bolyen et al., 2019), and amplicon sequence variants (ASVs) were inferred after quality filtering (Tedersoo et al., 2022; ASV count reported in Results). Single-end reads were imported from a manifest file and quality-filtered with quality-filter q-score (minimum quality 4, quality window 3, minimum length fraction 0.75, maximum ambiguous bases 0), then denoised with dada2 denoise-single (trunc-len 234, trim-left 0, max-ee 2.0, trunc-q 2, chimera-method consensus, independent pooling). Taxonomic classification used a naïve Bayes classifier trained on the UNITE fungal ITS database (v10.0, 2025-02-19) (Schoch et al., 2012; Nilsson et al., 2019; Abarenkov et al., 2025); the classifier was applied with feature-classifier classify-sklearn at a confidence threshold of 0.7, it was trained on the ITS2 region, and species-level assignments obtained from ITS2 alone are treated as provisional throughout. Features assigned to mitochondria or chloroplast, and features left unassigned at kingdom level, were then removed (taxa filter-table), and retained features were required to have a total frequency of at least one read (filter-features). This kingdom-level filter is distinct from the Unassigned fraction shown at family and genus level in **Supplementary Figure 2**, which consists of fungal reads that the ITS2 reference could not resolve to those ranks.All samples were rarefied to 7,800 reads prior to diversity analyses (McMurdie and Holmes, 2014); this threshold was set just below the smallest final library (7,868 reads after denoising, chimera removal and taxonomic filtering), so that 23 of 23 libraries were retained (final library sizes 7,868–23,269 reads, mean 15,567; **Supplementary Table 6**), and rarefaction depth is reported as a constraint of the exploratory microbiome analysis.The number of reads deposited in the Sequence Read Archive for each library ranged from 15,449 to26,871 (median 19,186; maximum/minimum ratio 1.74; **Supplementary Table 6**), so this depth was not imposed by a single unusually shallow deposit. The library that setsthe depth (GW_D_May_9; SRR38495249) is nevertheless the weakest of the 23 in processing terms,retaining 58.5% of its raw reads through quality filtering and 48.4% as non-chimeric against cohort means of 91.0% and 85.7% (**Supplementary Table 6**); it was retained because it is one member of a May–July tree pair used in the paired reanalysis and because its rarefaction curve is approaching plateau at 7,800 reads (**Supplementary Figure 5**). These are the read counts deposited for each run rather than post-denoising ampliconsequence variant table totals; the full per-sample DADA2 cascade (input, quality-filtered, denoised,non-chimeric and taxonomically filtered read counts, and observed ASVs) is given in **Supplementary Table 6**. Across the 23 libraries, 454,636 raw reads yielded 389,494 non-chimeric reads and 358, 046 reads after taxonomic filtering; per library, a mean of 91.0% of raw reads passed DADA2 quality filtering and 85.7% were retained as non-chimeric. Rarefaction curves are shown in **Supplementary Figure 5** and support this depth quantitatively: raising the depth from 6,000 to 7,800 reads added on average 2.1% observed ASVs (maximum 6.3%), and 20 of the 23 libraries gained less than 5%. Rarefaction was applied for alpha-diversity standardization, acknowledging the limitations of data-splitting (McMurdie and Holmes, 2014); compositionally aware analyses were therefore not deferred: Aitchison and robust-CLR distances were computed for the healthy-versus-diseased contrast, for which the Bray–Curtis result was the one carried into the Abstract, and are reported alongside, and given interpretive precedence over, that Bray–Curtis result. Bark scrape sampling was performed at the beginning of each monthly visit before the bead re-application scheduled for that visit; the May samples therefore represent the bark mycobiota approximately one month after the April baseline application, and the July samples represent approximately three months after the April application and two further monthly re-applications (in May and June).

No formal *post-hoc* power analysis was performed for the microbiome subset; instead, the permutation null distribution of the PERMANOVA R^2^ was used to bound what this design could detect. With these group sizes, an effect explaining less than 15.6% (Healthy versus May), 11.1% (May versus July), 17.4% (Healthy versus July) or 15.7% (three groups) of compositional variance could not have been declared significant at α = 0.05, so the observed May-versus-July R^2^ of 0.046 falls below the detection floor and that contrast is uninformative about differences smaller than roughly a tenth of the total variance, and the nominally significant Healthy-versus-July contrast (R^2^ = 0.178) exceeds its own detection floor of 17.4% only marginally; the microbiome analyses are accordingly presented throughout as exploratory baseline characterisation rather than as tests of a treatment effect; given the small group sizes (Healthy n = 4, May n = 9, July n = 10), non-significant microbiome comparisons should be interpreted as low-powered exploratory results rather than evidence of absence of a community effect.

**Figure 2:**
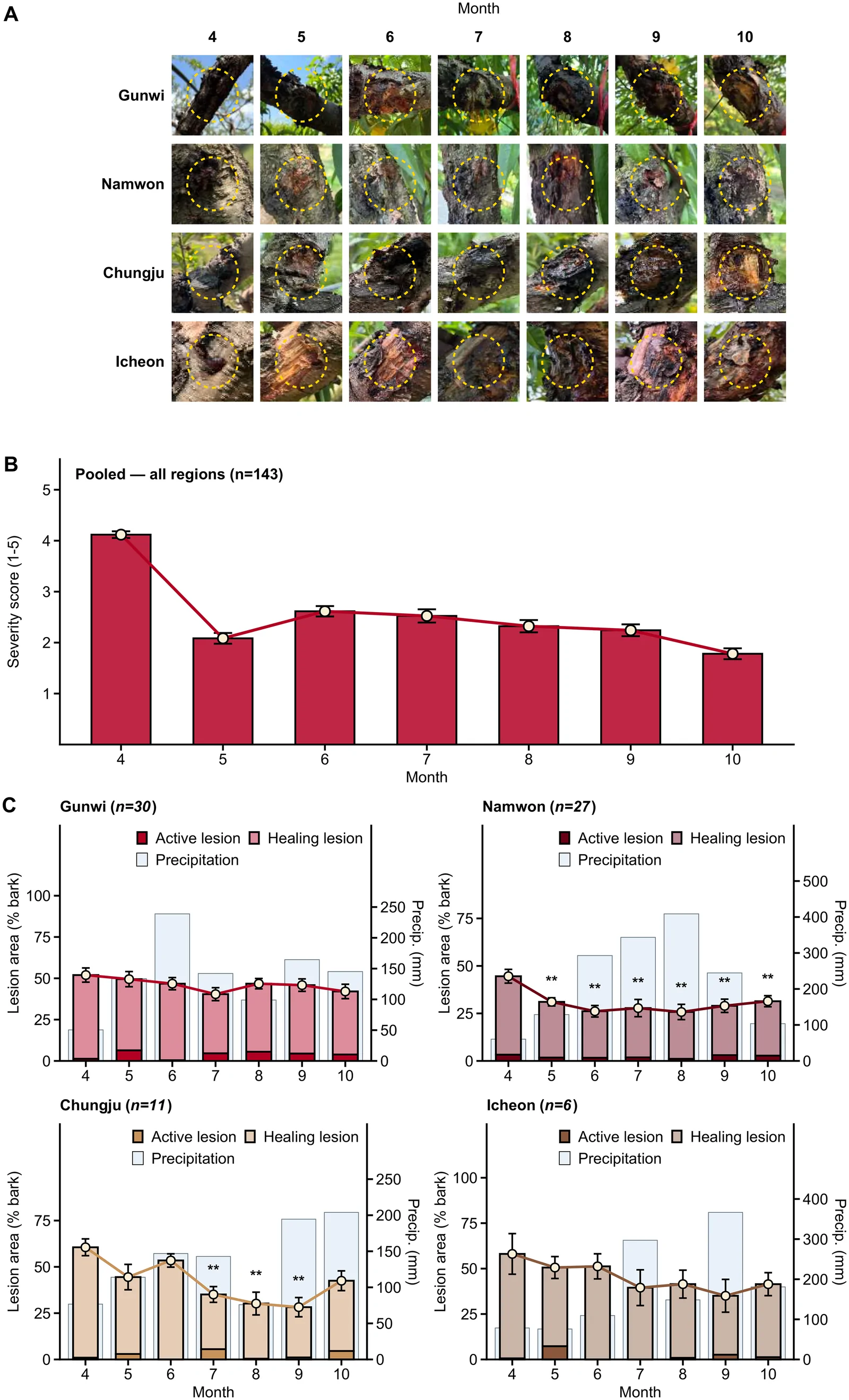
Multi-modal assessment of peach gummosis before and after application of a KNU-28-containing γ-PGA/alginate artificial biofilm across four study regions in 2025. **(A)** Algorithm-selected exemplar images (one of 74 cataloged trees per region; cohort-level statistical inference is based on the full 74-tree image set in panel C, not on these exemplars) illustrating lesion changes over time for one tree per region, Gunwi, Namwon, Chungju, and Icheon. Photographs were taken monthly from April (pre-treatment) to October. Representatives were selected from 74 cataloged trees using an automated pattern-score procedure favoring sequences that best illustrate heavy gummosis in April, a drop in symptom severity in May, and progressive scab formation from June to October; the highest-scoring sequence in each region is the one shown. Yellow dashed ellipses indicate the approximate location of the lesion. **(B)** Pooled field disease severity on an ordinal scale of 1–5, averaged across all four regions (n = 143 trees; mean ± SEM), which declined from the April baseline (4.12) to 2.32 in August (76.2% of trees improved; paired Wilcoxon signed-rank p = 1.5 × 10^–17^) and to 1.78 in October (three-region n = 128; 88.3%; p = 1.0 × 10^–19^). Per-region monthly severity trajectories, with regional precipitation and per-region significance testing, are shown in **Supplementary Figure 4**. **(C)** Image tile-based border-reference ΔE lesion quantifications based on all 74 trees, 30 in Gunwi, 27 in Namwon, 11 in Chungju, and 6 in Icheon (one tile per tree per month; 518 tiles in total). Bars show the mean per-tile lesion-associated fraction of bark-valid pixels, stacked into “active” (darker fill and wet, glossy amber gum) and “healing” (lighter fill and matte scab) components; error bars represent the SEM; lines represent the regional mean; and light-blue bars on the secondary axis represent the monthly precipitation. In panel C, significant differences between monthly means and April, based on one-sided paired Wilcoxon signed-rank tests with Benjamini–Hochberg false-discovery-rate correction applied within each region, are represented using asterisks—*p < 0.05; **p < 0.01; and ***p < 0.001—with “ns” indicating “not significant.” Region-level correlations between the monthly regional mean and the monthly regional precipitation are unadjusted exploratory values based on only seven monthly observations per region and reflect seasonal co-variation between post-treatment healing and the summer monsoon rather than a causal effect of moisture.

**Figure 3:**
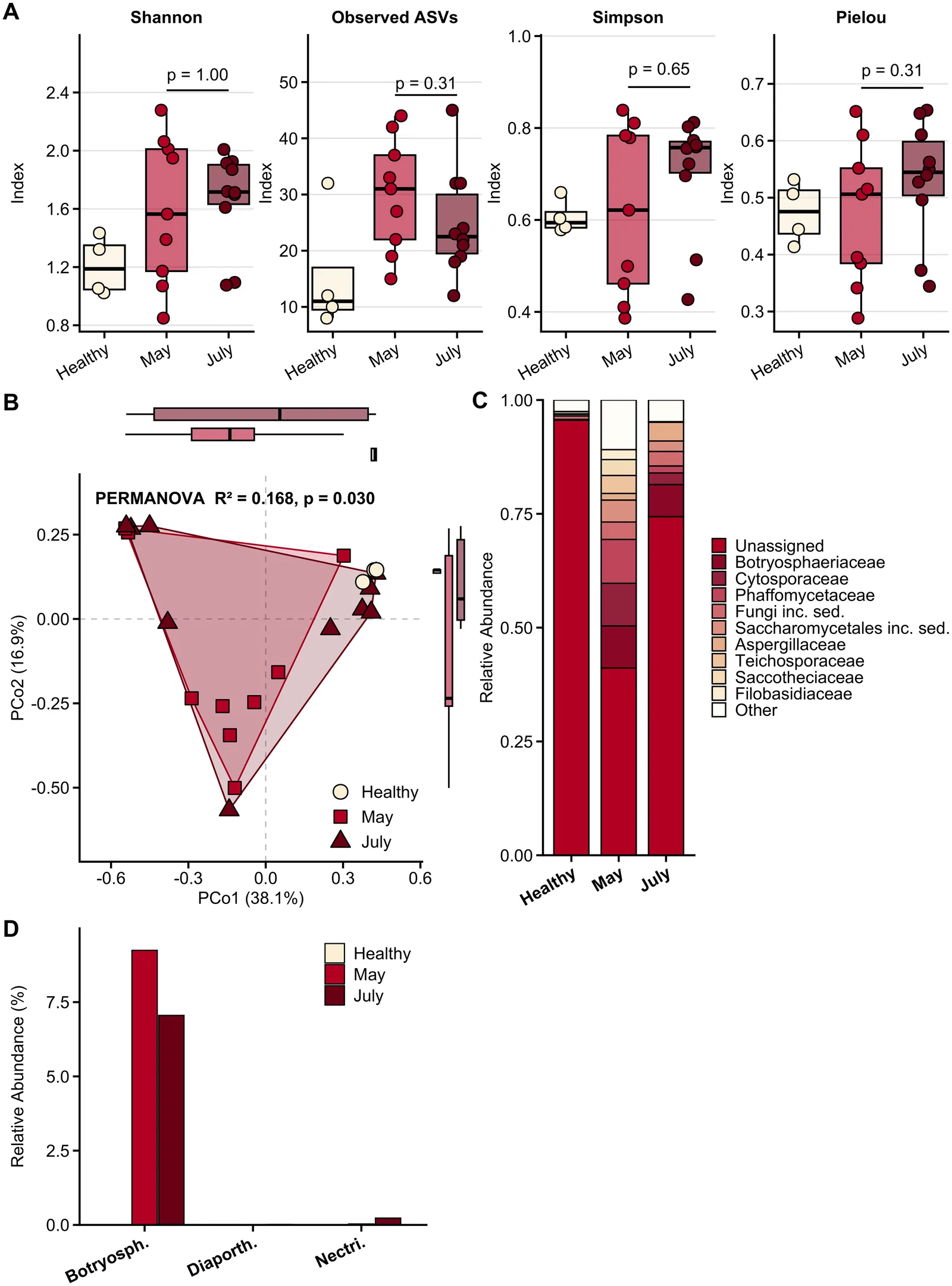
Overview of fungal microbiome diversity in peach bark samples across Healthy (n = 4), May (early treatment, n = 9), and July (later treatment, n = 10) groups. Panels show **(A)** alpha diversity comprising Shannon index, observed ASVs, Simpson’s index of diversity (1−D) and Pielou’s evenness J′, **(B)** Bray–Curtis dissimilarity-based PCoA, **(C)** family-level fungal community composition, and **(D)** selected pathogen-associated families. Panel **(A)** shows two-sided Mann–Whitney U tests comparing May (early treatment) and July (later treatment) groups. For panel **(B)**, multivariate dispersion differed significantly among the three groups (PERMDISP F = 20.7, p < 0.001), so the three-group PERMANOVA result on ASV-level Bray–Curtis dissimilarities (R^2^ = 0.168, p = 0.030) cannot be interpreted as a difference between group centroids; the separation was also not reproduced under compositionally aware distance metrics (see Discussion). These microbiome comparisons are exploratory because of the small sample size and unbalanced group structure.

**Figure 4:**
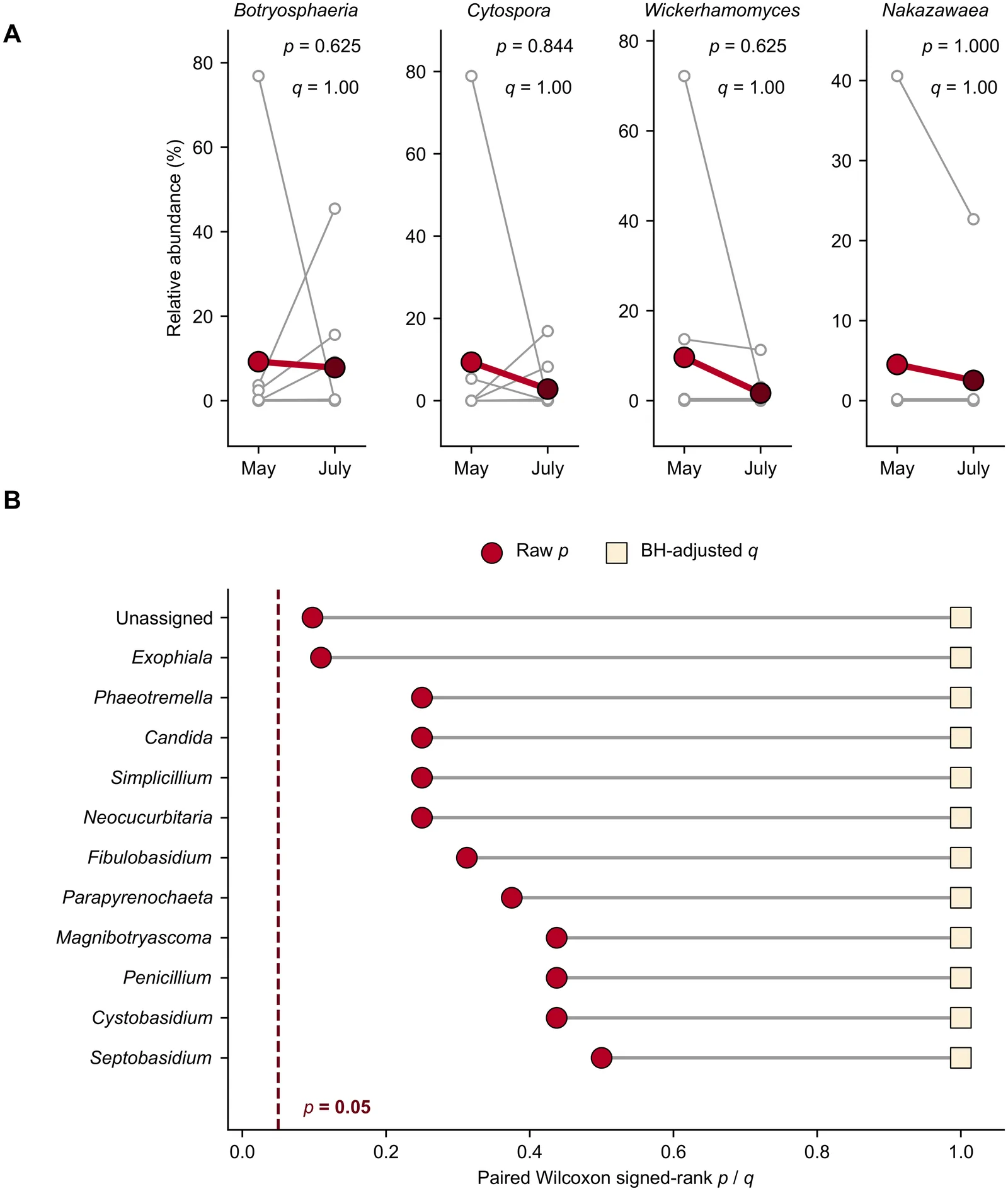
Paired reanalysis of the exploratory ITS2 dataset. **(A)** Paired trajectories for the nine trees sampled at both timepoints, one line per tree, with the paired Wilcoxon raw p and Benjamini–Hochberg q above each genus. **(B)** Raw p beside BH q for the twelve genera with the smallest raw p values; across all 71 genera tested in the nine matched trees the smallest raw p was 0.098 and every q equalled 1.00, so no genus differed between the two timepoints even nominally.

Alpha diversity (Shannon index, observed ASVs, Chao1 estimator, Simpson’s index of diversity (1−D), and Pielou’s evenness J′) was calculated in Python and compared across all three groups (Healthy, May and July) using two-sided Mann–Whitney U tests, with Benjamini–Hochberg correction applied across the full set of 15 contrasts (five metrics × three pairwise comparisons). Because 9 of the 10 diseased trees contributed libraries at both timepoints, the May-versus-July alpha comparison was additionally re-run as a paired Wilcoxon signed-rank test across those 9 matched trees. Phylogeny-aware distances (weighted and unweighted UniFrac) were not computed: the length variation of the ITS2 region across these fungal ASVs does not support an alignment we would regard as reliable for tree inference, and no phylogeny was therefore constructed. Beta diversity was assessed as Bray–Curtis dissimilarity computed at the amplicon sequence variant (ASV) level and visualized using principal coordinates analysis (PCoA), with group-level differences tested using a PERMANOVA with 9,999 permutations (Anderson, 2001; Anderson and Walsh, 2013) for both three-group and pairwise contrasts. Because PERMANOVA assumes homogeneity of multivariate dispersion, PERMDISP/betadisper was evaluated first and its outcome governs how the corresponding PERMANOVA result is interpreted: where dispersions were heterogeneous, the centroid contrast is not interpreted as a difference in community composition. Differential taxon abundances among groups were assessed with a custom log-ratio effect-size screen, which is distinct from and not equivalent to the LDA-based LEfSe algorithm (Segata et al., 2011): for each genus, a two-sided Mann–Whitney U test was applied on relative abundances and the group-mean effect size was computed as log_10_(mean_1_/mean_2_). A pseudocount of 0.5 reads (6.4 × 10^-5^ on the relative-abundance fraction scale, i.e. half the detection limit at a sequencing depth of 7,800 reads) was added to both group means before computing the log_10_ ratio, and genera with an |effect size| ≥ 1.5 were flagged. Because 9 of the 10 diseased trees contributed libraries at both timepoints, the Mann–Whitney component of this screen does not respect the paired structure of the May-versus-July contrast; for that contrast the screen is therefore used only to rank effect sizes, and inference rests on the paired Wilcoxon analysis reported in Section 3.6. Much smaller pseudocounts inflate the effect sizes of taxa detected in essentially only one group by several orders of magnitude and reorder the ranking; a sensitivity comparison across pseudocounts is provided in **Supplementary Figure 7**. Because no genus survived FDR correction under any pseudocount, this screen is reported as a qualitative exploratory ranking rather than as a test of differential abundance. Because we did not implement the original LDA-based classifier of LEfSe, we refer to this procedure hereafter as the “custom log-ratio effect-size” analysis to avoid conflation with the published LEfSe algorithm. Disease severity spanned almost no range within either sampling timepoint of the sequenced diseased libraries: the May field scores were 1–3 (median 2) and all ten trees scored 1 in July (the value of 5 recorded against the May libraries in the sequence-archive sample metadata is a group-level label applied to all diseased May samples, not a per-tree field score). Accordingly, “gummosis-affected” denotes the April enrolment status of these trees rather than their clinical state at the time of bark sampling: the sequenced trees scored 1–3 in May and 1 in July, the lowest category of the scale. In addition, 9 of the 10 contributing trees were sampled at both timepoints; correlating across these 19 libraries would therefore treat repeated measures on the same trees as independent observations. The genus–severity correlation analysis has accordingly been replaced by a paired comparison in which, for each genus, May and July relative abundances were compared across the 9 matched trees using a Wilcoxon signed-rank test; no correlation threshold was applied. Raw p values from all genus-level Mann–Whitney and paired Wilcoxon tests were adjusted for multiple testing using the Benjamini–Hochberg procedure, and q values (FDR) are reported alongside raw p values. All analyses were implemented in Python (SciPy; statsmodels for FDR adjustment). No analysis reported here was pre-registered; the pairwise and threshold-based contrasts — the regional Kruskal–Wallis tests, the girth correlations, and the effect-size and prevalence cut-offs — were specified after the data had been collected and are reported as *post-hoc* exploratory analyses. To address heterogeneity of multivariate dispersion as a confounder of PERMANOVA (Anderson and Walsh, 2013), we performed PERMDISP analyses on Bray–Curtis distances using the betadisper procedure of the R package vegan (v2.7.5), which tests the distances of each sample to its group spatial median in principal-coordinate space, with 9,999 permutations of the F-statistic. To assess robustness to distance-metric and compositionality assumptions, the PERMANOVA and PERMDISP analyses were repeated as sensitivity analyses using Aitchison distance (Euclidean distance on centered log-ratio (CLR)-transformed counts after addition of a pseudocount of 0.5), a robust CLR variant, and prevalence-filtered genus subsets. Compositional co-occurrence network inference (e.g., SparCC or SPIEC-EASI) was not attempted, because the 23-library exploratory subset is underpowered for stable network estimation; such ecological-interaction inference is deferred to a balanced, longitudinally resolved follow-up cohort. Final figures were rendered from the computed values described above without statistical recomputation: **Figures 2**–**4** and **Supplementary Figures 1, S2** were produced in R (v4.6.0) using the ggplot2 package (v4.0.3) with patchwork (v1.3.2) for multi-panel assembly, and **Figure 1** and **Supplementary Figure 3** were produced in Python using Matplotlib.

### 2.4 Tree girth and severity change correlation analysis

Stem circumference (girth; cm) was recorded at tree selection for each tree in Gunwi, Namwon, and Chungju (*n* = 128 trees). Girth was not measured in the Icheon region, and this region was excluded from this analysis. To assess whether tree size was associated with the observed change in severity, Spearman rank correlations were computed between girth and (i) baseline severity (April), (ii) final severity (October), and (iii) absolute change in severity (Δseverity = April − October) in Python using SciPy.

### 2.5 Image-based lesion-area quantification

To complement the semi-quantitative severity scoring with a measure of lesion burden whose quantification step is operator-independent — image acquisition itself was not blinded, and the residual bias this leaves is stated in the Limitations (point ix) — we used two pre-specified image sets. First, representative monthly photographs from four trees, one per region (Gunwi C09, Namwon A09, Chungju B05 and Icheon B05, top to bottom in **Figure 2A**), were used to illustrate the **Figure 2A** timeline panels; cohort-level statistical inference is based on the full 74-tree image set (**Figure 2C**), not on these individual exemplar trees. Second, the full cohort tile-based lesion-area quantification used all 74 photographed trees (30 in Gunwi, 27 in Namwon, 11 in Chungju, and 6 in Icheon; one bark tile per tree at each of the seven monthly visits, 518 tiles in total) and provided the cohort-level trajectories reported in **Figure 2C**. For each tile, lesion pixels were identified by a border-reference ΔE procedure: the outermost ≈13% border band of each tile was sampled as a per-tile healthy-bark colour reference, the tile was converted to CIE-LAB colour space, and pixels whose colour difference (ΔE, Euclidean distance in L*a*b*) from the border-reference median exceeded a fixed threshold of ΔE = 28 were retained as lesion candidates. Retained connected components were further restricted to those whose centroid lay within the central 10–90% of the tile in both axes, or that accounted for ≥20% of the detected lesion area. Lesion pixels were then classified as active (wet, glossy) or healing (matte, rough scab) using explicit HSV and texture criteria: a wet fraction (saturation > 100 with value between 95 and 220), a glossy fraction (value > 160), a dark fraction (value < 70) and the grey-level standard deviation were combined into an active score (wet fraction + 0.5 × glossy fraction) and a healing score (0.8 × dark fraction + 0.4 × grey-level standard deviation/50), and each component was assigned to the higher-scoring class. Background pixels were excluded by an upstream mask combining HSV and CIE-LAB rules (green foliage: hue 35–85 with saturation > 45; sky: hue 95–130 with saturation > 30; specular highlights: value ≥ 235; red tape used to label trees in the field: a* > 170 with value > 190). The resulting lesion mask was cleaned by morphological opening with a disk of radius 2 followed by closing with a disk of radius 3 (one iteration each), and connected components smaller than 800 pixels were discarded, a cut-off chosen to suppress isolated bark-texture speckle while retaining coherent lesions. Per-tile metrics included total lesion area, lesion fraction of the bark-valid area, particle count, and mean particle size; the border-reference ΔE threshold was established during method development by visual validation against a prototype panel of bark tiles and was then applied unchanged to the full 74-tree cohort; it was not re-tuned after inspecting cohort-level outcomes. Because the reference band can itself contain lesion tissue where a lesion reaches the tile edge, the detector was re-run on all 518 tiles with band widths of 8% and 20% alongside the 13% used throughout. Lesion masks intersected the reference band in 486 of 518 tiles (93.8%), and across the 500 tiles with detected lesion pixels the median overlap was 32.9% of lesion pixels, so contamination of the reference is common rather than exceptional; its effect is conservative, because widening the band lowered the mean lesion fraction from 39.9% to 37.8% while narrowing it raised the value to 41.5%, and tile-level values remained closely correlated across settings (r = 0.98 for 8% versus 13% and 0.97 for 20% versus 13%). The April-to-October regional declines were retained at every band width (−18% to −36%), so the cohort-level trajectory does not depend on this choice. Threshold parameters and per-tile metrics are provided as **Supplementary Data Sheet 1** (per-tile metrics for all 518 tiles) and **Supplementary Data Sheet 2** (the fixed pipeline parameters). The image measure was additionally validated against the independent field severity score (**Supplementary Figure 6**). Across 443 matched tree-month observations from the 65 photographed trees carrying unambiguous identifiers, agreement was statistically significant but weak (Spearman ρ = 0.16); mean lesion area rose from 34.4% at severity 1 to 44.4% at severity 4 but fell back to 41.1% at severity 5 (34.4, 42.3, 42.9, 44.4 and 41.1% for severity 1 to 5), so the relationship is not monotonic across the full scale (Kruskal–Wallis p = 0.003); discrimination between high (≥4) and low (≤2) severity was modest (AUC = 0.574); and the two measures agreed on the direction of the cohort-level seasonal decline but not on the ranking of individual trees (ρ = 0.15 between per-tree changes, p = 0.24). Tested separately within each month with Benjamini–Hochberg correction, the association reached significance only in May (ρ = 0.33, q = 0.049) and was marginal in October (ρ = 0.29, q = 0.09), and was not significant in the remaining five months (all q > 0.55). Twelve of the 455 possible tree-month observations could not be matched, all of them the six Icheon trees in September and October, because clinical severity scoring at Icheon was discontinued after August; the corresponding photographs were taken and are included in **Supplementary Data Sheet 1**. Two limits of this validation should be stated plainly. Trees scored 1, a category defined as showing no visible lesions, nevertheless returned a mean detected lesion fraction of 34.4%, so the measure indexes bark colour deviation from a per-tile local reference rather than a calibrated lesion area. No manually annotated ground truth was available, so the weak agreement is equally consistent with the two measures capturing different constructs and with limited validity of the detector itself. The image metric is therefore reported as a complementary and as yet unvalidated measure of bark lesion area rather than as an independent confirmation of the ordinal severity score, and the active/healing stratification, which was never compared against any clinical or annotated reference, is reported as descriptive only.

## 3 Results

### 3.1 Field response to artificial biofilm treatment

Across a 2.5–15% (w/v) γ-PGA concentration range, medium pH remained near-neutral (pH 6.93 → 6.90 → 6.83 → 6.81; ΔpH ≈ 0.12) while KNU-28 streak density on agar visibly declined at ≥10% γ-PGA, indicating, in this single-batch qualitative pilot screen, that the lower part of this range was the most suitable preliminary matrix window (**Supplementary Figure 3**; pilot screen, n = 1 per concentration). The γ-PGA-based artificial biofilm beads containing *Ba. amyloliquefaciens* KNU-28 were applied to peach trees with active gummosis in orchards across four regions, Gunwi (n = 50), Namwon (n = 38), Chungju (n = 40), and Icheon (n = 15), with a total of 143 trees sampled. At baseline (April), mean gummosis severity, assessed on a scale of 1–5, was uniformly high across regions: 4.18 in Gunwi, 4.08 in Namwon, 4.05 in Chungju, and 4.20 in Icheon (and at 4.12 for all samples pooled). By August, the last month with concurrent assessments in all four regions, regional mean scores had declined, to 2.14 in Gunwi, 2.84 in Namwon, 2.10 in Chungju, and 2.20 in Icheon, corresponding to a pooled mean of 2.32 (paired Wilcoxon April vs. August, W = 7685.5, p = 1.5 × 10^–17^, rank-biserial r = 0.86; **Figure 2**). Tree-level improvement, defined as an August severity score lower than that in April, was observed in 109 out of 143 trees overall (76.2%): 41 out of 50 (82.0%) in Gunwi, 25 out of 38 (65.8%) in Namwon, 32 out of 40 (80.0%) in Chungju, and 11 out of 15 (73.3%) in Icheon. The primary severity analysis was an ordinal cumulative-link mixed model with nested random intercepts (CLMM; n = 971 observations from 143 trees; R package ordinal, function clmm), which represents the repeated monthly observations as nested within trees and trees as nested within orchards; month was entered as a categorical fixed effect with April as the reference level, that specification having fitted substantially better than a linear time term (see below). Severity was lower than the April baseline at all six subsequent visits (all April-referenced contrasts p < 0.001), and under this specification the farm- and tree-level random-effect standard deviations were 0.366 and 0.717. In the secondary linear-month summary (severity ~ months_since_baseline + (1 | farm) + (1 | tree)) the odds of a tree occupying a higher severity category declined with time (β = −0.357 log-odds per month, SE = 0.032; z = −11.08; p = 1.5 × 10^-28^; 95% CI −0.420 to −0.294), corresponding to an odds ratio of 0.70 per month (95% CI 0.66–0.75). Both variance components were estimated simultaneously within this single model: the farm-level random-effect standard deviation was 0.335 and the tree-level random-effect standard deviation was 0.617 on the latent log-odds scale. Retaining the tree-level random intercept was statistically justified rather than assumed: the nested model improved on the farm-only specification both by likelihood-ratio test (χ^2^ = 19.23, df = 1, p = 1.16 × 10^-5^) and by AIC (2,851.3 versus 2,868.5), confirming that repeated observations on the same tree carry variance that a farm-level intercept alone does not absorb. Simpler specifications were retained only as sensitivity analyses and agreed in direction, magnitude and significance: single-random-effect CLMMs carrying the farm-level or the tree-level random intercept alone, a linear mixed-effects model on the 1–5 scale (β = −0.253 points per month, SE = 0.022; 95% CI −0.296 to −0.210; p < 0.001), a generalized estimating equation with exchangeable working correlation and cluster-robust standard errors clustered at the farm level (β = −0.253, robust SE = 0.028; z = −9.19; p < 0.001), and a generalized least-squares model with a first-order autoregressive within-tree correlation structure (β = −0.287, SE = 0.025; 95% CI −0.336 to −0.237; p < 0.001; AR(1) (Weaver, 1974) Φ = 0.258) (**Supplementary Table 4**). The estimated monthly time effect was therefore insensitive to the choice of model family and of within-cluster correlation structure. Because all of these specifications share a single linear monthly term, that agreement concerns the error and correlation structure rather than the functional form of time, which we therefore tested separately. A model treating month as a factor fitted substantially better than the linear term (AIC 2,733.6 versus 2,851.3; likelihood-ratio χ^2^ = 127.7, df = 5, p < 0.001), and a piecewise specification assigned most of the effect to the first inter-visit interval (April to May: β = −2.187, SE = 0.200, odds ratio 0.11) with a smaller but still significant decline thereafter (β = −0.139 per month, SE = 0.039, odds ratio 0.87, p < 0.001; AIC 2,761.4). The factor estimates were not monotonic, the June coefficient (β = −1.975) lying closer to the April baseline than the May coefficient (β = −2.845). Because the month-as-factor specification fits these data substantially better, it, rather than the linear term, is the description of the time course adopted here: relative to the April baseline the odds of occupying a higher severity category were May (0.058), June (0.139), July (0.106), August (0.079), September (0.075), October (0.031), every contrast p < 0.001. The monthly odds ratio of 0.70 should therefore be read as an average rate across the season rather than as a constant monthly rate (**Supplementary Table 4**). In the linear mixed model the farm-level intra-class correlation was small (ICC = 0.039); we do not read this as an absence of orchard-to-orchard heterogeneity, because the primary ordinal model estimates appreciable farm-level and tree-level random-effect standard deviations (0.366 and 0.717, respectively). The near-zero intra-class correlation is therefore an artefact of approximating an ordinal outcome with a linear-Gaussian model rather than evidence that orchard environments did not differ.

In Gunwi, Namwon, and Chungju, monthly assessments continued through October, and severity was lower in October than in April in each of them: regional mean severity reached 1.78 in Gunwi, 1.92 in Namwon, and 1.65 in Chungju by October, with a pooled mean of 1.78 (paired Wilcoxon April vs. October, W = 7061.5, p = 1.0 × 10^–19^, rank-biserial r = 0.95; **Supplementary Table 2**; **Supplementary Figure 4**). Tree-level improvement at the October endpoint was observed in 113 out of 128 trees (88.3%) across these three regions: 44 out of 50 (88.0%) in Gunwi, 34 out of 38 (89.5%) in Namwon, and 35 out of 40 (87.5%) in Chungju. The intervening trajectory was not monotonic: most of the change in these three regions occurred between the April and May visits (4.11 to 2.07), a partial rebound followed in June (2.63), and the mean then declined again to 1.78 by October; at Namwon the July mean (3.05) exceeded the May mean (1.53) (**Supplementary Table 2**). Because Icheon was clinically scored only through August, the October endpoint analysis is restricted to the three regions with full-season data.

Image-based border-reference quantification across the full cohort of 74 peach trees, 30 in Gunwi, 27 in Namwon, 11 in Chungju, and 6 in Icheon, with 518 tiles total (see the Methods), showed a seasonal decline concordant with the severity-score trajectories. Because agreement between the two measures at the level of individual trees is weak (Spearman ρ = 0.16; discrimination between high and low severity AUC = 0.574; **Supplementary Figure 6**), the image measure is reported as complementary to, rather than as an independent confirmation of, the ordinal score. Mean lesion areas were lower in October than in April in every region, although the intervening monthly trajectories were not monotonic (**Figure 2C**): from 52.0% to 42.0% in Gunwi (−19%), 44.6% to 31.4% in Namwon (−30%), 60.6% to 42.5% in Chungju (−30%), and 58.1% to 41.4% in Icheon (−29%, n = 6). We emphasize that the Icheon trajectory reported here for September and October (image-based lesion-area quantification: −29%, not statistically significant across six photographed trees) is an operator-independent, photograph-derived measure of bark lesion area and is conceptually and methodologically distinct from the five-stage clinical severity score; clinical severity scoring at Icheon was discontinued after August per the assessment schedule (see Methods) and the September–October clinical severity values are therefore not measured rather than null. The image-derived and clinical-severity trajectories at Icheon thus reflect not only different assessment windows but two distinct outcome measures, and the Icheon image-only September–October data should not be read as a clinical-severity finding. Month-by-month paired Wilcoxon tests against the April baseline (computed per tree on that tree’s single tile for the month; n = 74 trees), one-sided against the alternative of a decline and with Benjamini–Hochberg false-discovery-rate correction applied within each region, were significant at every assessed month in Namwon (all q < 0.01) and from July through September in Chungju (q < 0.01); the April-to-October lesion-area decline reached significance only in Namwon (q = 0.007), whereas Gunwi (q = 0.055), Chungju (q = 0.061), and Icheon did not reach significance after correction. The set of contrasts significant at q < 0.05 is unchanged if the same tests are computed two-sided. The absolute effect sizes are smaller than the severity-score reductions (48–59%), and between July and October the two measures diverged in direction, the pooled lesion-area fraction rising from 34.9% to 38.2% while the severity score continued to fall. Persistence of healed scar tissue in photographs after clinical resolution is one candidate explanation, but it was not tested here (**Figure 2C**). Stratification of lesion pixels into “active” (wet, glossy) and “healing” (matte, rough scab) classes did not resolve a treatment-associated transition: the healing class already accounted for 97.2% of detected lesion area at the pre-treatment April baseline, ranged between 89.5% and 98.2% at the subsequent visits, and stood at 90.6% in October, below its April value (**Supplementary Data Sheet 1**). This stratification is reported as descriptive only. Monthly regional mean lesion area was negatively associated with monthly regional precipitation in the two wettest sites, Namwon (ρ = −0.96, raw p < 0.001) and Icheon (ρ = −0.89, raw p = 0.007), but not in Gunwi (ρ = −0.61) or Chungju (ρ = −0.18) (both ns). These coefficients rest on only seven monthly observations per region, are reported as unadjusted exploratory values, and are correspondingly underpowered and susceptible to overfitting. We interpret them as seasonal co-variation between post-treatment healing and the summer monsoon rather than as a causal effect of moisture level, and we note that an uncontrolled design cannot separate seasonal from treatment-associated contributions.

### 3.2 Fungal alpha diversity

Fungal ITS2 amplicon libraries were prepared from 23 bark samples collected from a pre-specified subset of 11 ITS-sampled orchards (the Methods); these analyses are exploratory and were not designed to validate the severity findings from the full 18-farm severity cohort. A total of 238 ASVs were recovered from 23 fungal ITS amplicon libraries. Per-library read counts deposited in the Sequence Read Archive and the full per-sample DADA2 processing cascade, before rarefaction, are given in **Supplementary Table 6**. After rarefaction to 7,800 reads, Shannon diversity in the May group (1.594 ± 0.476) was comparable to that in the July group (1.663 ± 0.312; Mann–Whitney U = 45.0, p > 0.999, the value reflecting ties). Observed ASV richness also did not differ significantly between May (30.0 ± 9.5) and July (24.8 ± 8.8) samples (p = 0.307), nor did Chao1 richness (31.2 ± 10.7 in May and 25.7 ± 9.2 in July; p = 0.347). Simpson’s index of diversity (1−D) was similarly comparable between groups (May 0.621 ± 0.175 versus July 0.702 ± 0.122; p = 0.653), as was Pielou’s evenness J′ (0.472 ± 0.118 versus 0.530 ± 0.099; p = 0.307). All three group contrasts, including those against the healthy group, were tested for each of the five metrics, and none of the 15 comparisons remained significant after Benjamini–Hochberg correction (smallest q = 0.27; **Figure 3A**). Because 9 of the 10 diseased trees contributed libraries at both timepoints, treating the May and July libraries as independent would constitute pseudoreplication; the May-versus-July comparison was therefore also re-run as a paired Wilcoxon signed-rank test across those matched trees. Every metric available for the paired subset remained non-significant (Shannon raw p = 0.91; observed ASVs p = 0.27; Simpson’s index of diversity p = 0.50; Pielou’s evenness J′ p = 0.36; all q ≥ 0.66), so the unpaired result is not an artefact of the pairing structure. Given the group sizes (Healthy n = 4, May n = 9, July n = 10), these results indicate an absence of detectable differences in richness or evenness rather than evidence that alpha diversity was unaffected.

### 3.3 Beta diversity and community structure

The PCoA based on ASV-level Bray–Curtis dissimilarities showed apparent clustering among the three groups, with PC1 (38.1%) and PC2 (16.9%) together explaining 55.0% of total variance (**Figure 3B**). The healthy control-tree communities formed a cluster distinct from those of both gummosis groups along PC1, while May and July group samples showed partial separation along PC2.

Because PERMANOVA assumes homogeneity of multivariate dispersion, PERMDISP (betadisper) was evaluated first, and its outcome governs how the corresponding PERMANOVA is interpreted. On Bray–Curtis distances, multivariate dispersion was significantly heterogeneous across the three groups (F = 20.7, p < 0.001; betadisper, 9,999 permutations), with within-group dispersion markedly lower in healthy-tree communities (mean distance to the group spatial median 0.144) than in gummosis-affected communities (0.631 in May and 0.548 in July). The healthy-versus-May and healthy-versus-July contrasts were likewise heterogeneous (F = 127.3, p < 0.001, and F = 18.4, p = 0.002, respectively), whereas the May-versus-July contrast was not (F = 1.75, p = 0.20) (**Supplementary Table 5**). The heterogeneity is driven by the very small dispersion of the four healthy libraries relative to the diseased groups. Because dispersions are heterogeneous, a significant PERMANOVA cannot be interpreted as a difference between group centroids, and we therefore do not read the Bray–Curtis contrasts reported below as differences in community composition.

We accordingly treat the compositionally aware analyses as the primary beta-diversity result. For the healthy-versus-May contrast, neither Aitchison distance (Euclidean distance on centered log-ratio (CLR)-transformed genus counts with a pseudocount of 0.5; 87 genera; R^2^ = 0.089, p = 0.345) nor a robust CLR variant (87 genera; R^2^ = 0.083, p = 0.443) reproduced a significant separation, and the same held for every prevalence-filtered genus subset (prevalence ≥ 3/23, 36 genera, R^2^ = 0.049, p = 0.921; ≥ 6/23, 18 genera, R^2^ = 0.053, p = 0.804; ≥ 12/23, 5 genera, R^2^ = 0.063, p = 0.625); dispersion heterogeneity was also present under compositional distances (PERMDISP F = 6.42, p = 0.009 for Aitchison; F = 8.55, p = 0.003 for robust CLR) (**Supplementary Table 5**). The ASV-level Bray–Curtis PERMANOVA, reported for completeness in **Table 1**, returned nominal separation among the three groups (pseudo-F = 2.026, R^2^ = 0.168, p = 0.030), between healthy-tree and May communities (pseudo-F = 3.620, R^2^ = 0.248, p = 0.001) and between healthy-tree and July communities (pseudo-F = 2.599, R^2^ = 0.178, p = 0.048), whereas May and July communities did not differ (pseudo-F = 0.826, R^2^ = 0.046, p = 0.542). These pairwise p values are unadjusted exploratory contrasts; given the dispersion heterogeneity documented above and the absence of separation under every compositionally aware metric, they describe a Bray–Curtis dissimilarity pattern confounded by unequal within-group dispersion rather than a community-level difference.

**Table 1:** Results for the ASV-level Bray–Curtis dissimilarity-based PERMANOVA comparing fungal community compositions.

Comparison	Pseudo-F	R^2^	p (9,999 permutations)
Three groups (Healthy vs. May vs. July)	2.026	0.168	0.030*
Healthy vs. May (early treatment)	3.620	0.248	0.001**
Healthy vs. July (later treatment)	2.599	0.178	0.048*
May (early) vs. July (later)	0.826	0.046	0.542 ns

*p < 0.05; **p < 0.01; ns, not significant. p values are unadjusted and derived from PERMANOVA with 9,999 permutations.

### 3.4 Fungal community composition at the genus level

At the family level, gummosis-affected bark (May) showed higher relative abundances of Botryosphaeriaceae, Cytosporaceae and Phaffomycetaceae than healthy controls (**Figure 3C**). Among three pathogen-associated families that are well-recognised canker etiologic agents, Botryosphaeriaceae showed the highest relative abundance in gummosis-affected bark (observed group means: May 9.24%, July 7.05%), while Diaporthaceae and Nectriaceae remained near zero in all groups (**Figure 3D**). At the May sampling point, gummosis-affected peach bark was characterized by the co-dominance of four genera (**Table 2**), *Wickerhamomyces* (at 9.64% relative abundance), *Cytospora* (at 9.36%), *Botryosphaeria* (at 9.24%), and *Nakazawaea* (at 4.53%), all of which were absent or negligible in healthy control trees. Among these co-detected genera, *Wickerhamomyces anomalus*, like other fruit-associated yeasts in the Saccharomycetales that have been investigated for biocontrol of postharvest fungal pathogens (Masih and Paul, 2002), is recognized as a biocontrol-associated taxon rather than a peach canker pathogen; its high relative abundance in gummosis-affected bark but absence from healthy trees is consistent with opportunistic colonization of bark that carried active gummosis lesions at enrolment rather than a causal role in disease. *Cytospora* is the causal agent of *Cytospora* canker, a bark canker disease distinct from *Botryosphaeria*-driven gummosis; its co-detection alongside *Botryosphaeria* at comparable relative abundances indicates that fungi associated with at least two different canker diseases were present simultaneously in these trees. Observed group mean relative abundances at the two diseased sampling points are reported side by side in **Table 2** (*Wickerhamomyces* May 9.64% and July 1.53%; *Cytospora* 9.36% and 2.55%; *Botryosphaeria* 9.24% and 7.05%; *Nakazawaea* 4.53% and 2.29%; *Penicillium* 1.46% and 4.11%). None of these May-versus-July differences was statistically significant (raw p 0.125–0.909; all Benjamini–Hochberg q > 0.5; **Table 2**). These values are reported as descriptive group means only, and no directional or comparative interpretation of the genus-level patterns is offered. No individual taxon retained significance after Benjamini–Hochberg adjustment (all q > 0.5). Because the 19 diseased libraries derive from only 10 trees, 9 of which were sampled at both timepoints, the comparison was additionally re-run as a paired test across those 9 matched trees. No genus differed even nominally (71 genera tested; smallest raw p = 0.098; all q = 1.00; **Figures 4A, B**), and the nominal reduction in *Fibulobasidium* returned by the unpaired test (0.95% to 0.46%, raw p = 0.030) did not persist when the pairing was respected (paired raw p = 0.31). At the ASV level, the single dominant *Botryosphaeria* amplicon sequence variant — which accounted for essentially all genus-level *Botryosphaeria* reads in gummosis-affected (May) bark (≈9.2% of the 9.24% genus-level total) — was classified under the UNITE reference database as *Botryosphaeria qingyuanensis* rather than *B. dothidea*, whereas the co-dominant *Cytospora* was resolved only to the genus level (its most abundant ASV was unassigned at species level, with minor variants matching *Cytospora vinacea*). Because ITS2 naïve-Bayes classification is not a substitute for multilocus species delimitation, these read-based assignments narrow, but do not finalize, the species identity of the canker-associated taxa (see Limitations).

**Table 2:** Relative abundances (%) of dominant fungal genera across sample groups, with May vs. July Mann–Whitney U test raw p values and Benjamini–Hochberg false-discovery-rate q values (computed across all tested genera).

Genus	Healthy (%)	May (%)	July (%)	Δ (July − May)	May vs. July raw *p*	BH q
*Wickerhamomyces*	0.00	9.64	1.53	−8.11	0.899	> 0.5
*Cytospora*	0.15	9.36	2.55	−6.81	0.586	> 0.5
*Botryosphaeria*	0.00	9.24	7.05	−2.19	0.860	> 0.5
*Nakazawaea*	0.00	4.53	2.29	−2.24	0.909	> 0.5
*Fibulobasidium*	0.00	0.95	0.46	−0.50	0.030	> 0.5
*Penicillium*	0.00	1.46	4.11	+2.65	0.125	> 0.5
*Aureobasidium*	0.47	3.49	0.07	−3.43	0.146	> 0.5
*Simplicillium*	0.02	0.00	0.01	+0.01	0.094	> 0.5
*Rhodotorula*	0.00	0.01	0.16	+0.16	1.000	> 0.5
*Alternaria*	0.41	0.22	0.26	+0.04	0.730	> 0.5
*Cladosporium*	0.21	0.16	0.35	+0.19	0.336	> 0.5
*Botrytis*	0.04	0.00	0.00	+0.00	1.000	> 0.5
*Chaetocapnodium*	0.88	0.00	0.00	+0.00	1.000	> 0.5
*Hyphodontia*	0.08	0.00	0.01	+0.01	0.399	> 0.5
*Diaporthe*	0.00	0.00	0.01	+0.01	0.193	> 0.5

The delta symbol, “Δ,” represents the change in relative abundance between the early (May) and later (July) treatment timepoints (July – May). No individual genus reached q < 0.05.

### 3.5 Custom log-ratio effect-size screen: exploratory ranking

We next identified genera that discriminated among groups using a custom log-ratio effect-size screen (Mann–Whitney U test combined with a log_10_(mean_1_/mean_2_) effect size computed with a 0.5-read pseudocount; see Methods). Because no genus survived FDR correction in any contrast, and because the ranking produced by this screen depends on the pseudocount chosen, it is presented as an exploratory ranking only and the full results are given in **Supplementary Figure 7**.

Healthy control group vs. May group: Twenty genera exceeded the |effect size| ≥ 1.5 threshold. Among these, *Wickerhamomyces* (effect size = −3.18), *Botryosphaeria* (−3.16), and *Nakazawaea* (−2.85) showed the largest effects, all of them reflecting a higher mean relative abundance in May gummosis-affected bark. Only two genera exceeded the threshold in the opposite direction, *Chaetocapnodium* (+2.14) and *Aspergillus* (+1.55). No genus was significant after Benjamini–Hochberg correction (smallest q = 0.524) (**Supplementary Figure 7**).

May group vs. July group: Six genera exceeded the |effect size| ≥ 1.5 threshold, and none exceeded it with the opposite sign: *Naganishia* (2.06), *Kwoniella* (1.88), *Magnibotryascoma* (1.80), *Aureobasidium* (1.69), Pleosporales gen. Incertae sedis (1.59) and *Calosphaeria* (1.53) (**Supplementary Figure 7**). Consistent with the non-significance of the May-versus-July PERMANOVA contrast, no individual genus-level Mann–Whitney U test was significant after Benjamini–Hochberg FDR correction (smallest q = 0.599). This screen moreover compared the May and July libraries as unpaired groups even though 9 of the 10 contributing trees were sampled at both timepoints, so pseudoreplication remains within it; the ranking is exploratory only, it is superseded by the paired reanalysis across the matched trees (**Figure 4**), and no directional interpretation of the May-versus-July genus patterns is offered.

### 3.6 Correlation between disease severity and fungal genera

Disease severity varied over at most three adjacent categories within the sequenced diseased libraries (May 1–3, median 2; July 1 in all ten trees), and 9 of the 10 contributing trees were sampled at both timepoints; treating each library as an independent observation would therefore constitute pseudoreplication. The correlation analysis reported previously has accordingly been withdrawn in favour of the paired comparison described above. In that analysis no genus differed between the two timepoints even nominally (smallest raw p = 0.098; all q = 1.00; **Figure 4B**), including every taxon previously highlighted: *Fibulobasidium* (paired raw p = 0.31), *Simplicillium* (p = 0.25), *Penicillium* (p = 0.44), *Aureobasidium* (p = 0.56), *Candida* (p = 0.25) and *Septobasidium* (p = 0.50). No genus is therefore identified here as a candidate marker of disease severity or of disease suppression.

### 3.7 Tree girth and severity change

To examine whether tree size was associated with the observed change in severity, a Spearman rank correlation analysis assessing the relationship between stem girth and gummosis severity metrics was performed across the three regions in which girth was recorded (Gunwi, Namwon, and Chungju; *n* = 128 trees). Girth at tree selection was not correlated with baseline severity (ρ = −0.032, *p* = 0.72), indicating that trees of different sizes experienced comparable initial disease severity. Girth showed a weak negative correlation with final (October) severity (ρ = −0.194, raw p = 0.028) and no association with Δseverity (April severity score – October severity score; ρ = 0.140, raw p = 0.11); because Δseverity is computed from the same April and October scores, it is not statistically independent of the two correlations reported above and is not treated as separate evidence for a tree-size effect. After Benjamini–Hochberg correction across the three correlations, none was significant (q = 0.72, 0.085 and 0.17, respectively). These associations are of small magnitude (|ρ| < 0.2) and do not support a tree-size effect on the observed severity change; the cohort is also underpowered for a size-stratified analysis.

## 4 Discussion

### 4.1 Field response and scope of inference

In this uncontrolled pre/post cohort, treatment of peach trees with the KNU-28-containing artificial biofilm was associated with a pronounced seasonal decline in gummosis severity across all four regions, with overall severity in pooled samples dropping from 4.12 in April to 2.32 by August, and 76.2% of the 143 monitored trees showed a severity score improvement of at least one point. Additionally, scores continued to decline, falling to 1.78 by October, the end of the growing season, in Gunwi, Namwon, and Chungju, with 88.3% of 128 trees showing improvement. Although the magnitude of the within-tree severity decline observed here falls within the range reported for *Bacillus*-based biocontrol formulations in stone-fruit systems (Li et al., 2016; Kang et al., 2024), the absence of a concurrent untreated control arm in the present study—unlike those comparators—precludes any direct, quantitative head-to-head efficacy comparison; we therefore frame the present results as a platform-level field demonstration rather than as a competing efficacy estimate. Whether the γ-PGA matrix causally contributed to this decline cannot be established from an uncontrolled design; the data are compatible with, but do not prove, a contribution of matrix-mediated persistence of the biocontrol agent on the bark surface by protecting cells from desiccation and UV exposure while maintaining close contact with bark tissue (Guijarro et al., 2019; Saberi Riseh et al., 2021).

The γ-PGA-based artificial biofilm used here was designed against a documented constraint: biocontrol bacteria applied to plant surfaces often survive poorly, so that conventional spray-based formulations provide only short-lived protection (Saberi Riseh et al., 2021). It is designed to mitigate these losses through three complementary material and ecological properties. First, γ-PGA is an anionic, highly hygroscopic biopolymer whose water-retention and mild pH-buffering behavior is expected to attenuate the acidic, dehydrating microenvironment characteristic of active gummosis lesions, providing conditions compatible with KNU-28 cell survival on bark surfaces (John et al., 2011; Ogunleye et al., 2015). Second, γ-PGA serves not only as a passive carrier but also contributes to *Bacillus* biofilm formation and matrix cohesion, supporting surface adhesion and the slow release of viable cells (Vlamakis et al., 2013; Yu et al., 2016); the Ooho-like bead format therefore presents KNU-28 to the bark in a state that is biophysically pre-organized for biofilm-like persistence rather than as free planktonic cells. Third, biofilm-embedded cells are generally more tolerant of environmental stressors than planktonic cells (John et al., 2011; Vlamakis et al., 2013), an attribute relevant to survival on exposed bark through the summer monsoon and into October. While none of these properties was directly measured *in situ* in the present field cohort — KNU-28 colonization dynamics were not quantified by strain-specific qPCR, a limitation already acknowledged in the Limitations — the observed severity trajectory cannot bear on them, because an uncontrolled pre/post design cannot separate a treatment contribution from seasonality, concurrent orchard management, regression to the mean and assessor expectation. A hypothesized encapsulation-mediated extension of the operational window of the biocontrol agent therefore remains untested here, and it motivates dedicated persistence assays in follow-up controlled trials.

### 4.2 γ-PGA biofilm as a generalizable delivery platform

Beyond the specific KNU-28–gummosis pairing examined here, the γ-PGA-based Ooho-like biofilm is, by design, agent-agnostic: it encapsulates viable bacterial cells in a biodegradable, bark-adhesive matrix without relying on any property unique to KNU-28. The same material rationale that motivated its use here — γ-PGA’s hygroscopic water retention, its contribution to *Bacillus* biofilm formation (Vlamakis et al., 2013; Yu et al., 2016), and the environmental-stress tolerance of biofilm-embedded cells (John et al., 2011; Vlamakis et al., 2013) — applies in principle to other *Bacillus* and PGPR biocontrol agents and to other bark- or surface-borne plant diseases. We therefore frame the formulation as a candidate delivery system for field application of bacterial biocontrol agents; however, because no carrier-only (KNU-28-free) control arm was included in this demonstration, the formulation’s field performance relative to a carrier-only control remains to be established. The γ-PGA matrix’s suitability as a delivery system for other agents or other crops is a design-property hypothesis that requires validation in dedicated controlled trials (see Future work).

Three features of this study constrain the strength of causal inference. First, because no untreated gummosis-affected control trees were employed—a design choice rather than an unavoidable constraint, since a randomly selected subset of the enrolled trees could have been left untreated—we cannot separate biofilm-attributable recovery from background seasonal dynamics, such as summer bark suberization, reduced pathogen sporulation during dry periods, or variation in annual weather patterns. The trees also remained under routine commercial management throughout the observation window. Because the growers’ spray programmes, irrigation and pruning were not recorded, any contribution of concurrent management to the observed decline can neither be estimated nor excluded; it is a co-intervention rather than a background descriptive variable, and it stands alongside seasonality and regression to the mean among the explanations that an uncontrolled pre/post design cannot separate from the treatment. The gummosis pathogen’s annual infection cycle is seasonal, so part of the observed trajectory probably reflects natural seasonality rather than treatment alone. The analogy with pistachio, in which the same broad-sense pathogen infects in summer and autumn and expresses symptoms the following spring (Michailides, 1991), is indicative only, because *Botryosphaeria* species differ in life cycle between hosts and the cycle on peach in these orchards was not characterised. Second, severity scores are based on visual lesion assessment and are not independent of treatment status. Formal efficacy estimates will require randomized, blinded trials with matched untreated controls. Furthermore, because trees were enrolled on the basis of high baseline disease severity (April pooled mean 4.12 on a 1–5 scale), a regression-to-the-mean effect—whereby extreme initial measurements tend to return towards the population average at subsequent assessments regardless of intervention—is expected and cannot be quantified without a concurrent untreated control arm (Bland and Altman, 1994). Bounding this contribution numerically would require assumed values for the untreated population mean and for test–retest reliability, neither of which an uncontrolled design can supply; the resulting range is correspondingly wide and uninformative, and we therefore do not report one. Regression to the mean cannot be formally excluded as a partial contributor to the observed decline. This is a limit on what a pre/post design without concurrent controls can establish, not a quantity that the present data can estimate.

Third, because the same trained personnel applied treatments and recorded field scores, assessor expectation bias cannot be excluded. Blinding was limited to prior-month scores, and no independent untreated arm or formal inter-rater reliability analysis was available to separate measurement variability from true temporal change; the operator-independent image-based quantification (see Methods) was included specifically to provide a treatment-status-independent measure less susceptible to this expectation bias.

Tree stem girth showed only weak associations with the observed change in severity, e.g., with severity in October (ρ = −0.194, raw p = 0.028; q = 0.085 after Benjamini–Hochberg correction), Δseverity (ρ = 0.140, raw p = 0.11), and baseline severity (ρ = −0.032, *p* = 0.72). The magnitude of these effects (|ρ| < 0.2) is small relative to the overall severity decline, and thus, we do not interpret these correlations as suggesting increased treatment benefit for mature trees. A larger cohort allowing a size-stratified analysis would be required to resolve whether bark surface area, rootstock age, or induced-resistance capacity meaningfully modulate biofilm performance.

### 4.3 Exploratory microbiome patterns associated with disease severity

Multivariate dispersion was significantly heterogeneous among the bark groups under both Bray–Curtis (PERMDISP F = 20.7, p < 0.001) and CLR distances (PERMDISP p < 0.01), and heterogeneous dispersions preclude interpreting a PERMANOVA centroid contrast as a difference in community composition. Under compositionally aware distances (Aitchison/CLR distance, a robust CLR variant, and prevalence-filtered subsets), the healthy-versus-diseased contrast was not statistically significant (R^2^ ≈ 0.05–0.09, p = 0.345–0.921). Against that background, the nominal separation of healthy from gummosis-affected (May) bark returned by ASV-level Bray–Curtis PERMANOVA (R^2^ = 0.248, p = 0.001; three-group p = 0.030) is not interpreted here as a compositional difference. The heterogeneity reflects the very small dispersion of the healthy group (mean distance to the group spatial median 0.144) relative to the diseased groups (0.631 in May and 0.548 in July). We therefore treat the healthy-versus-diseased separation as a Bray–Curtis-detectable pattern that is confounded by dispersion heterogeneity and is not reproduced under compositionally aware metrics, rather than as a community-level difference. The dominance of *Botryosphaeria*, *Wickerhamomyces*, *Cytospora*, and *Nakazawaea* in diseased trees (**Table 2**) is consistent with pathogen overgrowth and opportunistic colonization of previously lesioned bark, but, given the metric sensitivity and dispersion heterogeneity noted above, should be regarded as an exploratory descriptive pattern in this small subset rather than a confirmed community restructuring (Anderson and Walsh, 2013).

The non-significance of the May-versus-July PERMANOVA centroid contrast (p = 0.542) should be interpreted cautiously given the modest sample sizes (n = 9 and n = 10). No individual genus retained significance after Benjamini–Hochberg adjustment (all q > 0.5), and in the paired reanalysis across the nine matched trees none differed even nominally. The apparent genus-level differences between the two timepoints in the four canker-associated taxa (*Botryosphaeria*, *Cytospora*, *Wickerhamomyces*, *Nakazawaea*) and in *Penicillium* are therefore statistically unsupported in this dataset, and we do not interpret them as community restructuring, as recovery toward a healthy-bark state, or as a treatment effect.

Whether biofilm treatment measurably reshapes the diseased bark mycobiota is a hypothesis that must be tested in a balanced, longitudinally resolved cohort that includes an untreated diseased control and strain-level KNU-28 tracking; the present data serve only to generate that hypothesis. Accordingly, we frame the microbiome component of this study as a field-scale characterization of the diseased-versus-healthy bark phytobiome (Philippot et al., 2013; Vacher et al., 2016) — a baseline prerequisite for interpreting future treatment effects (see Introduction) — rather than as evidence of a treatment-driven community shift.

### 4.4 Genera highlighted by the exploratory effect-size screen

Among the genera highlighted by the custom log-ratio effect-size screen, none differed between the two timepoints after Benjamini–Hochberg correction or in the paired reanalysis across the nine matched trees, and several occur at very low or near-trace relative abundance (for example, *Simplicillium* and *Diaporthe* each at ≤0.02% in the relevant sample groups; **Table 2**), so their large log-ratio effect sizes are driven by the presence or absence of rare taxa rather than by graded abundance shifts. Although *Simplicillium*, *Penicillium* and *Aureobasidium* species are documented producers of antifungal metabolites and have been examined as biocontrol agents in stone-fruit systems and against Botryosphaeriaceae canker pathogens (Shin et al., 2017; Pinto et al., 2018; Guijarro et al., 2019; Li et al., 2023), none is advanced here as a biocontrol candidate: the severity correlations on which such a suggestion would have rested have been withdrawn as pseudoreplicated, and the paired reanalysis found no genus-level difference even nominally. For *Aureobasidium*, the apparent group-level difference rests on its near-absence from most gummosis-affected libraries together with a single high-abundance May outlier (~31%) that inflates the May group mean (**Table 2**), so mean- and rank-based summaries diverge and it should not be over-interpreted. One caveat nevertheless warrants explicit statement: *Diaporthe*, one of the genera detected in this bark community, encompasses several established woody-plant canker and dieback pathogens (e.g., *D. eres* and *D. ambigua*), so any future change in this bark community should not be assumed to be uniformly beneficial across the wider canker-pathogen pool, and the genus warrants dedicated targeted-sampling follow-up in a powered cohort.

*Fibulobasidium* was highlighted in the previous version of this analysis on the basis of a genus–severity rank correlation that has since been withdrawn as pseudoreplicated (see Results). In the paired reanalysis across the nine matched trees its relative abundance did not differ between the two timepoints even nominally (paired raw p = 0.31; q = 1.00), and it is therefore not advanced here as a disease-associated taxon.

### 4.5 Hypothesized mechanisms

*Bacillus amyloliquefaciens* KNU-28 may be associated with reduced gummosis through multiple, currently hypothesized mechanisms that remain unverified in the present field cohort: (i) the direct antagonism of *Bo. dothidea* via the production of lipopeptide antibiotics like iturin A, fengycin, and surfactin, as suggested by dual-culture inhibition assays (**Figure 1C**) and consistent with the lipopeptide biosynthetic gene clusters characteristic of the operational group *Ba. amyloliquefaciens* (Chen et al., 2007; Fan et al., 2017); this inference is, however, indirect, because the dual-culture assay was performed against the *Bo. dothidea* reference isolate KACC45481, whereas the dominant *Botryosphaeria* amplicon sequence variant recovered from these orchards was assigned to *Bo. qingyuanensis*, so antagonism of the *Botryosphaeria* population actually present in the field has not been tested; (ii) the competitive exclusion of the gummosis pathogen by occupying bark surface niches; and (iii) the potential induction of systemic resistance in the host plant (Bais et al., 2004; Ongena et al., 2007). The γ-PGA matrix might contribute through moisture retention, physical wound coverage, or the creation of a favorable micro-environment for KNU-28 persistence, but none of these mechanisms was tested here: they are hypotheses drawn from the literature and from the design of the formulation rather than findings of this study; however, because KNU-28 colonization of treated bark was not directly quantified in this study (see Limitations, point iii), we cannot distinguish KNU-28-mediated activity from matrix-only effects or background seasonal recovery. No shift in fungal community composition is demonstrated here: no genus differed between the two timepoints after Benjamini–Hochberg correction or in the paired reanalysis across the nine matched trees. Whether KNU-28 application produces broader microbiome-level changes beyond the direct effects of the strain itself therefore remains an untested hypothesis rather than an observation, and requires controlled trials with an untreated diseased arm.

### 4.6 Limitations and caveats

Several features of this demonstration trial warrant caution in the interpretation of its results: (i) There was no untreated control. As noted above, the pre-treatment/post-treatment design cannot separate biofilm effects from the natural seasonal dynamics of peach gummosis; thus, observed declines likely include a background seasonal component. (ii) The assessment window was unbalanced across regions. Gunwi, Namwon, and Chungju were monitored monthly from April through October, while Icheon was scored clinically from April through August, with monthly photographs for the image analysis continuing through October. The primary four-region pooled analysis is, therefore, based on the April–August window common to all sites, but the October endpoint was used only for the three regions with data for the full season. (iii) KNU-28 colonization was not measured. We did not quantify KNU-28 establishment on treated bark using strain-specific qPCR or amplicon-based tracking, so the extent and duration of its persistence *in situ* is unknown. (iv) The microbiome sample sizes were small and collected over a short period. Sampling for community analysis was restricted to 23 bark samples collected at two time points, and larger, longitudinally resolved cohorts will be required to test whether community restructuring is robust and attributable to the biofilm biocontrol treatment. In addition, the healthy comparator trees (Healthy, n = 4) were not matched to the diseased trees—they were sampled from partly different orchards (n = 4 versus 19) and were, by definition, trees that had not developed gummosis—so the healthy-versus-diseased community difference may reflect pre-existing compositional differences or reverse causation rather than a disease-induced change, and is interpreted throughout as a cross-sectional disease-state association. (v) No genus-level result survived multiple-testing correction. Benjamini–Hochberg adjustment rendered every genus-level comparison non-significant, and a paired reanalysis across the nine matched trees found no genus differing even nominally; raw p values are reported for transparency only. Statements about individual genera are therefore hypothesis-generating at most.

Additional limitations are that (vi) the γ-PGA concentration screen was a qualitative single-batch pilot (n = 1 per concentration), so it supports formulation feasibility rather than a statistically validated optimum; (vii) the near-zero farm-level intra-class correlation returned by the linear mixed model (ICC = 0.039) is an artefact of approximating an ordinal outcome with a linear-Gaussian model and must not be read as a biological absence of orchard-to-orchard heterogeneity: the primary ordinal model estimates farm-level and tree-level random-effect standard deviations of 0.366 and 0.717 respectively (**Supplementary Table 4**), which are not negligible; (viii) patent protection limits disclosure of the exact formulation sequence and timing, although academic non-commercial access to the material can be requested through Microbalance Inc. and the corresponding author under an MTA; (ix) the principal investigator served simultaneously as treatment applicator, primary severity assessor, and a financial stakeholder in Microbalance Inc. (the patent holder and commercial licensor of the KNU-28 formulation), which leaves a residual pathway for unconscious assessor bias; formal inter-rater reliability statistics could not be computed because only consensus field scores were recorded, and independent third-party severity assessment was not implemented. The operator-independent image measure mitigates but does not remove this pathway, because the same personnel selected and photographed the trees and because its agreement with the ordinal score at the level of individual trees was weak (**Supplementary Figure 6**); (x) the 19 diseased libraries derive from only 10 trees, 9 of which contributed at both timepoints; the severity-correlation analysis that treated these libraries as independent observations has been withdrawn and replaced by a paired test across the matched trees; (xi) the monthly rate of severity decline was positively correlated with temperature variables (**Supplementary Figure 1**; ρ = +0.44–0.52, uncorrected), so part of the apparent treatment-associated improvement during the warm season may reflect temperature-accelerated natural bark recovery rather than biofilm action and cannot be statistically separated within this uncontrolled design; and (xii) molecular identification of the causal pathogen was not performed in the current study; the species assignment *Bo. dothidea* follows historical records from these orchards and should be understood as *Bo. dothidea sensu lato* (see the Introduction, where this is disclosed, and refs 3 and 5). Consistent with this, ITS2 amplicon profiling of diseased bark in the present study assigned the dominant *Botryosphaeria* variant to *Bo. qingyuanensis* under the UNITE reference database — indicating that the gummosis-associated *Botryosphaeria* population in these orchards may not correspond to *Bo. dothidea sensu stricto* — and species-level attribution of these canker pathogens therefore requires multilocus (ITS, *tef1*, *tub2*) confirmation in future work. A direct consequence is that the only direct antagonism evidence presented here (**Figure 1C**) was obtained against the *Bo. dothidea* reference isolate KACC45481 rather than against the *Botryosphaeria* taxon that dominated these orchards, so antagonism of the field population remains undemonstrated. Finally, (xiii) a number of study-descriptive variables requested during review are not reported here: they were not compiled for this study, and we prefer not to reconstruct them retrospectively. These are the rootstock and age of the enrolled trees; per-orchard management history (irrigation, pruning and fungicide use, both concurrent with and prior to the trial; the concurrent component is treated as a co-intervention in Section 4.2); the weather conditions on individual application days; storage conditions and holding time for the formulation between preparation and application; and post-encapsulation viable-cell counts, which were not enumerated. For the same reason, the content and duration of the assessors’ training is not reported, and no formal biological or technical replicate structure is reported for the field applications, so replication in this demonstration is at the level of trees and orchards only. The bark-sampling procedure is likewise not reported in the detail requested during review: the scraping depth, the position of each scrape relative to the lesion margin, the number of scrapes pooled per tree and the bead-beating conditions applied during DNA extraction were not compiled for this study and are therefore not reported here. Relatedly, the five-stage severity scale specifies lesion diameter and lesion count jointly, and no written precedence rule was prespecified for trees in which the two criteria indicated different categories; such cases were resolved by consensus between the two assessors at the time of scoring, and the absence of a documented rule is a further reason to treat the ordinal scores as semi-quantitative. The DADA2 per-sample denoising statistics have since been retrieved from the sequencing facility’s QIIME 2 provenance records and are reported in full in **Supplementary Table 6**; because the libraries were sequenced single-end, no read-merging step applies and no merging parameters exist to report.

### 4.7 Practical implications

This study provides field-scale uncontrolled pre/post evidence that application of the KNU-28-containing artificial biofilm coincided with reduced peach gummosis severity in commercial peach orchards. The biodegradable γ-PGA formulation is designed to address a key barrier to the adoption of biocontrol agents—namely, poor field persistence—although persistence on bark was not measured in this study, and it is intended to meet demands for environmentally safe crop protection. The formulation has been licensed for commercial development (Daewon Chemical Co., Ltd.), and the present field data suggest that controlled scale-up evaluation is warranted, but registration-oriented efficacy claims will require randomized untreated-control trials and strain-level persistence measurements.

Future work should include (i) re-testing KNU-28 antagonism against *Botryosphaeria* isolates recovered from these orchards — in particular *Bo. qingyuanensis*, the taxon to which the dominant field amplicon sequence variant was assigned — because the present dual-culture evidence was obtained against a *Bo. dothidea* reference isolate; (ii) randomized controlled trials with untreated controls; (iii) application timing and dose–response optimization; (iv) evaluations in cherry and plum orchards; (v) the quantitative characterization of KNU-28 colonization dynamics on bark using strain-specific qPCR, CFU recovery from bark, fluorescent labeling and colonization imaging to establish persistence and dose–exposure relationships; and (vi) balanced, adequately powered microbiome cohorts that would enable compositional co-occurrence network inference (e.g., SparCC or SPIEC-EASI), which the present 23-library exploratory subset cannot support.

We emphasize that the present study is a multi-orchard demonstration/hypothesis-generating trial without concurrent no-treatment controls; therefore, the observed severity declines and the exploratory bark-mycobiome trajectories are reported as treatment-associated rather than as formal causal-efficacy estimates. A randomized, blinded field trial with matched untreated-control trees—ideally paired within the same orchard block to absorb spatially structured background variability—will be required before formal efficacy or registration claims can be made on behalf of the KNU-28 artificial-biofilm formulation.

## 5 Conclusion

A γ-PGA-based artificial biofilm encapsulating *Ba. amyloliquefaciens* KNU-28 was applied across commercial peach orchards in four administrative regions of South Korea and coincided with within-tree seasonal declines in gummosis severity in an uncontrolled multi-orchard demonstration trial, with 76.2% of 143 monitored trees showing improvement in disease symptoms relative to the April baseline by August and 88.3% of 128 trees in the three regions followed through October reaching a lower severity by season’s end. In parallel, fungal community profiling showed significantly heterogeneous multivariate dispersion between gummosis-affected and healthy bark (PERMDISP p < 0.001) and no separation under compositionally aware distance metrics, so the nominal ASV-level Bray–Curtis PERMANOVA separation (p = 0.001) cannot be read as a compositional difference; it therefore provides an exploratory microbial baseline for this disease that requires confirmation in a powered, balanced cohort. Apparent differences between the early- (May) and later-treatment (July) diseased communities were non-significant (p = 0.542; all genus-level q > 0.5). No directional interpretation of these genus-level patterns is therefore offered. Because disease severity varied over at most three adjacent categories in the sequenced subset and most trees contributed samples at both timepoints, the genus–severity correlations reported previously have been withdrawn in favour of a paired analysis, in which no genus differed even nominally (smallest raw p = 0.098; all q = 1.00). Tree size was not strongly associated with the observed change in severity. Together, these findings identify the KNU-28-containing artificial biofilm as a candidate for controlled evaluation in the field management of peach gummosis, warranting follow-up randomized trials with untreated controls and strain-level colonization tracking before formal efficacy claims can be made. More broadly, the γ-PGA-based Ooho-like biofilm is presented as a candidate agent-agnostic delivery platform for bark-associated biocontrol — a design property that itself requires dedicated validation — and the operator-independent border-reference ΔE image workflow offers a parameter-transparent method for quantifying canker lesion area from field photographs, whose agreement with clinical assessment was weak at the level of individual trees and whose active/healing stratification remains unvalidated.

## Data Availability

The raw ITS amplicon sequencing data generated in this study are deposited in the NCBI Sequence Read Archive (SRA) under BioProject accession PRJNA1464532 (https://www.ncbi.nlm.nih.gov/bioproject/PRJNA1464532). Individual BioSample accessions (SAMN59720672–SAMN59720694) and SRA Run accessions (SRR38495228–SRR38495250) for all 23 libraries are provided in **Supplementary Table 3**, and the data are mirrored at the European Nucleotide Archive through the INSDC partnership.

## Supplementary material

Supplementary Figure 1 Spearman rank correlation analysis of the relationship between monthly weather variables, including mean temperature, precipitation, relative humidity, and sunshine duration, and gummosis severity across four regions (April–October 2025). **(A)** Heatmap of Spearman correlation coefficients between eleven weather variables and gummosis severity (top row, absolute monthly severity) and the month-to-month change in severity (bottom row). **(B)** Monthly disease severity (solid line, left axis), mean temperature (dashed line, right axis), and precipitation (bars) for each region. **(C)** Scatter plots of the five weather variables significantly correlated with absolute severity (pooled across regions, n = 26): sunshine, diurnal temperature range, wind speed, humidity, and rainy days. **(D)** Scatter plots of the five weather variables significantly correlated with the month-to-month change in severity (n = 22): ground temperature, maximum temperature, precipitation, mean temperature, and minimum temperature. Significance is indicated as **p* < 0.05; ***p* < 0.01; ****p* < 0.001.

Supplementary Figure 2 Taxonomic composition of bark fungal communities across Healthy (n = 4), May (early treatment, n = 9), and July (later treatment, n = 10) groups. Stacked bars show mean relative abundance at the **(A)** phylum, **(B)** family, and **(C)** genus level. Top taxa at each rank are shown explicitly; remaining taxa are pooled into Other. All data are based on rarefied read counts (7,800 reads per sample). The high Unassigned fraction in panels **(B)** and **(C)** reflects ITS2 amplicon sequences that could not be resolved to those taxonomic levels.

Supplementary Figure 3 *In vitro* screening of the γ-PGA matrix concentration range used in the artificial biofilm formulation. *Bacillus amyloliquefaciens* KNU-28 was zigzag-streaked on 1/10-strength LB agar (1.5% w/v agar) supplemented with γ-PGA at 2.5%, 5%, 10%, or 15% (w/v) and incubated at 30 °C for 22 h (n = 1 per concentration; single-batch pilot). **(A)** Medium pH measured immediately after autoclaving and plate pouring across the four γ-PGA concentrations; values remained near-neutral (pH 6.93–6.81; ΔpH ≈ 0.12 across the 6-fold concentration range), indicating that γ-PGA does not appreciably acidify the medium under 1/10-strength LB buffering. **(B–E)** Representative streaking plate photographs at 2.5%, 5%, 10%, and 15% γ-PGA, respectively; visible colony density and zigzag streak opacity decline progressively at ≥10% γ-PGA despite stable medium pH, consistent with a non-pH-mediated viability constraint at high matrix concentrations. The lower part of the screened range (2.5–5% w/v) was therefore retained as the preliminary working range for the Ooho-form formulation described in the Methods.

Supplementary Figure 4 Per-region monthly gummosis severity. Field disease severity on an ordinal scale of 1–5 (1 = no visible lesions/complete recovery; 5 = severe disease) for each of the four study regions: Gunwi (Daegu; n = 50), Namwon (Jeonbuk-do; n = 38), Chungju (Chungcheongbuk-do; n = 40), and Icheon (Gyeonggi-do; n = 15; not scored in September–October). Bars represent monthly means ± SEM and lines represent the regional mean trajectory; light-blue bars on the right-hand secondary axis represent the monthly regional total precipitation (mm). Significant differences between each monthly mean and the April baseline, based on paired Wilcoxon signed-rank tests with Benjamini–Hochberg correction across the six monthly contrasts, are indicated by asterisks (*, *p* < 0.05; **, *p* < 0.01; ***, *p* < 0.001). The pooled all-region trajectory is shown in **Figure 2B**.

Supplementary Figure 5 Rarefaction curves for the 23 ITS2 libraries. Observed ASVs are plotted against sequencing depth and coloured by group, with the rarefaction depth of 7,800 reads indicated. Raising the depth from 6,000 to 7,800 reads added on average about 2% observed ASVs, and 20 of the 23 libraries gained less than 5%, supporting 7,800 reads as the common depth.

Supplementary Figure 6 Validation of the border-reference ΔE image measure against the independent field severity score. **(A)** Lesion-area fraction by severity score (Kruskal–Wallis p = 0.003). **(B)** Within-month Spearman correlations with Benjamini–Hochberg q values; only May reached significance (q = 0.049). **(C)** Per-tree change in severity against per-tree change in lesion area (ρ = 0.15, p = 0.24, n = 65; the two measures moved in the same direction in 41 of the 65 trees, 63.1%). (D) Region-by-month means (ρ = 0.39, p = 0.050, n = 26). Agreement between the two measures is weak at the level of individual trees, although both decline over the season at cohort level.

Supplementary Figure 7 Sensitivity of the custom log-ratio effect-size screen to the pseudocount. Effect sizes for the healthy-versus-May and May-versus-July contrasts were computed with pseudocounts of 1 × 10^-9^, 0.5 reads (6.4 × 10^-5^) and 0.5%. The ranking of the May-versus-July contrast changes materially with the pseudocount, and no genus is significant after FDR correction under any of the three values (smallest q = 0.524 and 0.599).

Supplementary Data Sheet 1 Per-tile output of the border-reference ΔE lesion-quantification pipeline for all 518 tiles (74 photographed trees × seven monthly visits, April–October 2025).

Supplementary Data Sheet 2 Fixed parameters of the border-reference ΔE detection and active/healing classification pipeline, as applied to the full 74-tree cohort without re-tuning.

SUPPLEMENTARY TABLE 1 Geographic coordinates (latitude/longitude), administrative-region assignments and enrolled tree counts for the 18 commercial peach orchards participating in the 2025 KNU-28 artificial-biofilm field trial.

SUPPLEMENTARY TABLE 2 Region-level monthly mean disease severity scores for the 143-tree cohort across Gunwi, Namwon, Chungju, and Icheon (April-October 2025), with per-region sample sizes and standard errors of the mean. Per-region monthly significance testing against the April baseline is shown in **Supplementary Figure 4**.

SUPPLEMENTARY TABLE 3 BioSample and SRA Run accessions for the 23 ITS amplicon libraries associated with BioProject PRJNA1464532.

SUPPLEMENTARY TABLE 4 Summary statistics for the nested ordinal cumulative-link mixed models - the primary specification with month as a categorical fixed effect and the secondary linear-month summary (CLMM; severity ~ months_since_baseline + (1 | farm) + (1 | tree)) - and the sensitivity analyses: single-random-effect CLMMs carrying the farm-level or the tree-level random intercept alone, the linear mixed-effects model (LMM), the generalized estimating equation (GEE; cluster-robust SE clustered at the farm level), and the generalized least-squares model with a first-order autoregressive within-tree correlation structure (AR(1)).

SUPPLEMENTARY TABLE 5 PERMDISP (betadisper) analysis of Bray-Curtis distance multivariate dispersion across the three bark sample groups (Healthy, May, and July).

SUPPLEMENTARY TABLE 6 Per-library read counts and per-sample DADA2 processing statistics for the 23 ITS2 amplicon libraries (BioProject PRJNA1464532).
