# Supplementary material for: A γ-polyglutamic acid-based artificial biofilm for field delivery of Bacillus amyloliquefaciens KNU-28: an uncontrolled multi-orchard demonstration on peach gummosis with exploratory bark fungal community profiling
Source: Front Plant Sci. 2026 Aug 31;17:1933566. doi: 10.3389/fpls.2026.1933566 (PMC13572429; doi:10.3389/fpls.2026.1933566)
Supplement: Supplementary file 1 [file Table1.docx]

**Supplementary Table S1.** Farm-level study sites and tree counts.

Locations and enrolled tree counts for the 18 commercial peach orchards used in the field trial (143 trees in total; Gunwi 50, Namwon 38, Chungju 40, Icheon 15). Coordinates are the GPS positions recorded in the field for each surveyed orchard block; exact farm names are withheld at the request of participating growers.

| **Region (Province)** | **Farm code** | **Latitude (°N)** | **Longitude (°E)** | **n (trees)** |
| --- | --- | --- | --- | --- |
| Gunwi (Daegu) | GW_A | 36.10190 | 128.71464 | 10 |
| Gunwi (Daegu) | GW_B | 36.14228 | 128.70906 | 10 |
| Gunwi (Daegu) | GW_C | 36.13701 | 128.69514 | 10 |
| Gunwi (Daegu) | GW_D | 36.24688 | 128.53966 | 10 |
| Gunwi (Daegu) | GW_E | 36.24630 | 128.54594 | 5 |
| Gunwi (Daegu) | GW_F | 36.24823 | 128.55099 | 5 |
| Namwon (Jeonbuk-do) | NW_A | 35.34870 | 127.35202 | 10 |
| Namwon (Jeonbuk-do) | NW_B | 35.36698 | 127.35151 | 10 |
| Namwon (Jeonbuk-do) | NW_C | 35.35992 | 127.27465 | 10 |
| Namwon (Jeonbuk-do) | NW_D | 35.41628 | 127.33704 | 8 |
| Chungju (Chungcheongbuk-do) | CJ_A | 37.11103 | 127.76772 | 10 |
| Chungju (Chungcheongbuk-do) | CJ_B | 37.10334 | 127.75612 | 10 |
| Chungju (Chungcheongbuk-do) | CJ_C | 37.11138 | 127.74993 | 10 |
| Chungju (Chungcheongbuk-do) | CJ_D | 37.07414 | 127.75637 | 5 |
| Chungju (Chungcheongbuk-do) | CJ_E | 37.04886 | 127.75143 | 5 |
| Icheon (Gyeonggi-do) | IC_A | 37.11381 | 127.60927 | 5 |
| Icheon (Gyeonggi-do) | IC_B | 37.12071 | 127.61256 | 5 |
| Icheon (Gyeonggi-do) | IC_C | 37.13764 | 127.61895 | 5 |

*Farm codes follow the convention {Region prefix}_{Farm letter}: GW = Gunwi, NW = Namwon, CJ = Chungju, IC = Icheon. Tree counts are the number of gummosis-affected peach trees enrolled at the April baseline visit.*

Note: bark samples for the fungal community profiling (Supplementary Table S3) were collected at 11 of these 18 orchards (GW_A, GW_C, GW_D, GW_E, GW_F, NW_B, CJ_A, CJ_D, CJ_E, IC_A and IC_C); all 18 orchards contributed treated and monitored trees to the severity cohort.

**Supplementary Table S2.** Regional mean disease severity scores by month (April–October 2025).

Mean ± SEM gummosis severity scores on a five-stage scale (1 = no visible lesions; 5 = lesion diameter > 5 cm with > 10 lesions), computed across trees within each region for every monthly visit. Icheon was clinically scored from April through August only; monthly photographs for the image analysis continued through October.

| **Region** | **n** | **April** | **May** | **June** | **July** | **August** | **September** | **October** |
| --- | --- | --- | --- | --- | --- | --- | --- | --- |
| Gunwi | 50 | 4.18 ± 0.11 | 2.26 ± 0.20 | 2.34 ± 0.19 | 2.34 ± 0.19 | 2.14 ± 0.19 | 2.08 ± 0.16 | 1.78 ± 0.18 |
| Namwon | 38 | 4.08 ± 0.13 | 1.53 ± 0.18 | 2.66 ± 0.20 | 3.05 ± 0.27 | 2.84 ± 0.26 | 2.68 ± 0.24 | 1.92 ± 0.20 |
| Chungju | 40 | 4.05 ± 0.13 | 2.35 ± 0.18 | 2.98 ± 0.16 | 2.33 ± 0.25 | 2.10 ± 0.21 | 2.02 ± 0.20 | 1.65 ± 0.17 |
| Icheon | 15 | 4.20 ± 0.20 | 2.20 ± 0.26 | 2.47 ± 0.26 | 2.33 ± 0.45 | 2.20 ± 0.40 | — | — |
| Pooled (4 regions, Apr–Aug) | 143 | 4.12 ± 0.07 | 2.08 ± 0.11 | 2.62 ± 0.10 | 2.52 ± 0.13 | 2.32 ± 0.12 | — | — |
| Pooled (3 regions, Apr–Oct) | 128 | 4.11 ± 0.07 | 2.07 ± 0.11 | 2.63 ± 0.11 | 2.55 ± 0.14 | 2.34 ± 0.13 | 2.24 ± 0.12 | 1.78 ± 0.11 |

*Em-dash indicates that the corresponding visit was not performed. Values are computed directly from the per-tree field record (n = 143 trees); SEM is the standard error of the mean across trees.*

**Supplementary Table S3.** BioSample and SRA Run accessions for the 23 ITS amplicon libraries.

All sequencing data are deposited under NCBI BioProject PRJNA1464532. Treatment group abbreviations: Healthy = healthy control bark (May 2025); May = early-treatment gummosis-affected bark (May 2025); July = later-treatment gummosis-affected bark (July 2025).

| **Sample ID** | **Treatment group** | **Region** | **BioSample (SAMN)** | **Run (SRR)** |
| --- | --- | --- | --- | --- |
| GW_D_May_8 | May | Gunwi | SAMN59720672 | SRR38495250 |
| GW_D_May_9 | May | Gunwi | SAMN59720673 | SRR38495249 |
| GW_D_Jul_8 | July | Gunwi | SAMN59720674 | SRR38495238 |
| GW_D_Jul_9 | July | Gunwi | SAMN59720675 | SRR38495234 |
| GW_E_May_5 | May | Gunwi | SAMN59720676 | SRR38495233 |
| GW_F_May_3 | May | Gunwi | SAMN59720677 | SRR38495232 |
| NW_B_May_3 | May | Namwon | SAMN59720678 | SRR38495231 |
| GW_E_Jul_5 | July | Gunwi | SAMN59720679 | SRR38495230 |
| GW_F_Jul_3 | July | Gunwi | SAMN59720680 | SRR38495229 |
| NW_B_Jul_3 | July | Namwon | SAMN59720681 | SRR38495228 |
| CJ_D_May_5 | May | Chungju | SAMN59720682 | SRR38495248 |
| CJ_E_May_1 | May | Chungju | SAMN59720683 | SRR38495247 |
| CJ_D_Jul_5 | July | Chungju | SAMN59720684 | SRR38495246 |
| CJ_E_Jul_1 | July | Chungju | SAMN59720685 | SRR38495245 |
| CJ_E_Jul_4 | July | Chungju | SAMN59720686 | SRR38495244 |
| IC_A_May_2 | May | Icheon | SAMN59720687 | SRR38495243 |
| IC_C_May_3 | May | Icheon | SAMN59720688 | SRR38495242 |
| IC_A_Jul_2 | July | Icheon | SAMN59720689 | SRR38495241 |
| IC_C_Jul_3 | July | Icheon | SAMN59720690 | SRR38495240 |
| GW_A_May_ctrl | Healthy | Gunwi | SAMN59720691 | SRR38495239 |
| GW_C_May_ctrl | Healthy | Gunwi | SAMN59720692 | SRR38495237 |
| CJ_A_May_ctrl | Healthy | Chungju | SAMN59720693 | SRR38495236 |
| IC_A_May_ctrl | Healthy | Icheon | SAMN59720694 | SRR38495235 |

*Each Sample ID is linked to its BioSample and SRA Run accession using the run metadata retrieved from the NCBI Sequence Read Archive for BioProject PRJNA1464532; the accessions listed here are the assignments recorded by NCBI and are not inferred from submission order. Per-library read counts are given in Supplementary Table S6.*

**Supplementary Table S4. Primary ordinal cumulative-link mixed model and sensitivity analyses of the decline in gummosis severity over time.**

All models test the within-tree decline in gummosis severity score as a function of months_since_baseline, accounting for clustering of repeated measurements. The nested ordinal cumulative-link mixed model (CLMM) carrying both a farm-level and a tree-level random intercept is the primary analysis and reports the time effect on the log-odds scale; the single-random-effect CLMMs, the linear mixed-effects model (LMM), the generalized estimating equation (GEE) and the generalized least-squares model with a first-order autoregressive within-tree correlation structure are sensitivity analyses. The farm-level and tree-level random-effect standard deviations of the primary model are estimated simultaneously within that single model and are therefore components of one variance decomposition. The small farm-level intra-class correlation of the linear model (ICC = 0.039) is an artefact of approximating an ordinal outcome with a linear-Gaussian model and does not indicate an absence of orchard-to-orchard heterogeneity.

| **Model** | **Effect** | **β (SE)** | **95% CI** | **z** | **p** | **Random effect** | **AIC** |
| --- | --- | --- | --- | --- | --- | --- | --- |
| CLMM, ordinal, (1 \| farm) + (1 \| tree) — primary (R package ordinal, clmm) | months_since_baseline (log-odds) | −0.357 (0.032) | −0.420 to −0.294 | −11.08 | 1.5 × 10⁻²⁸ | farm SD = 0.335; tree SD = 0.617 | 2,851.3 |
| CLMM, ordinal, (1 \| farm) — sensitivity | months_since_baseline (log-odds) | −0.338 (0.031) | −0.399 to −0.277 | −10.87 | < 0.001 | farm SD = 0.348 | 2,868.5 |
| CLMM, ordinal, (1 \| tree) — sensitivity | months_since_baseline (log-odds) | −0.357 (0.032) | −0.420 to −0.294 | −11.08 | < 0.001 | tree SD = 0.711 | 2,855.1 |
| LMM (severity ~ months + (1 \| farm); ML) — sensitivity | months_since_baseline | −0.253 (0.022) | −0.296 to −0.210 | −11.57 | < 0.001 | farm ICC = 0.039 | 3,350.9 |
| GEE (exchangeable, cluster-robust by farm) — sensitivity | months_since_baseline | −0.253 (0.028) | −0.307 to −0.199 | −9.19 | < 0.001 | — | — |
| GLS with AR(1) within tree — sensitivity | months_since_baseline | −0.287 (0.025) | −0.336 to −0.237 | −11.42 | < 0.001 | AR(1) Φ = 0.258 | 3,313.4 |
| CLMM, ordinal, month as factor — sensitivity | month (factor; April = reference) | May −2.845 (0.238); June −1.975 (0.220); July −2.244 (0.233); Aug −2.534 (0.236); Sep −2.585 (0.238); Oct −3.461 (0.258) | May −3.310 to −2.379; Oct −3.965 to −2.956 | −13.44 to −8.99 | < 0.001 (LRT vs. linear χ² = 127.7, df = 5, p < 0.001) | farm SD = 0.366; tree SD = 0.717 | 2,733.6 |
| CLMM, ordinal, piecewise — sensitivity | April→May step; per month thereafter | −2.187 (0.200); −0.139 (0.039) | −2.579 to −1.794; −0.216 to −0.063 | −10.93; −3.57 | 8.6 × 10⁻²⁸; 3.6 × 10⁻⁴ | farm SD = 0.349; tree SD = 0.694 | 2,761.4 |

*n = 971 observations from 143 trees in 18 farms. The LMM, GEE and AR(1) models are reported on the original severity scale (1–5); the primary CLMM β is on the log-odds scale, where β = −0.357 corresponds to an odds ratio of 0.70 per month (95% CI 0.66–0.75). Retaining the tree-level random intercept was supported against the farm-only specification by a likelihood-ratio test (χ² = 19.23, df = 1, p = 1.16 × 10⁻⁵) and by AIC (2,851.3 versus 2,868.5). The ordinal and autoregressive models were fitted in R (packages ordinal and nlme); the LMM and GEE were fitted in Python (statsmodels). AIC values are not comparable across the linear-Gaussian and ordinal frameworks. The final two rows test the functional form of the time term rather than the error structure: treating month as a factor fits substantially better than the linear term (ΔAIC = 117.7), and a piecewise fit assigns most of the effect to the first inter-visit interval, so the linear monthly odds ratio of 0.70 is an average rate across the season rather than a constant monthly rate.*

**Supplementary Table S5. PERMDISP (betadisper) tests of multivariate homogeneity of dispersion.**

Tests performed on Bray–Curtis distances with the betadisper procedure of the R package vegan (v2.7.5), using 9,999 permutations of the F-statistic computed from the distance of each sample to its group spatial median in principal-coordinate space. These values were recomputed for this revision and supersede those in the originally submitted table (response letter, item N7).

| **Comparison** | **Groups** | **F** | **p** | **Mean distance to centroid (per group)** |
| --- | --- | --- | --- | --- |
| Three-group overall | Healthy / May / July | 20.73 | < 0.001 | Healthy 0.144; May 0.631; July 0.548 |
| May vs. July | May / July | 1.75 | 0.197 | May 0.631; July 0.548 |
| Healthy vs. May | Healthy / May | 127.34 | < 0.001 | Healthy 0.144; May 0.631 |
| Healthy vs. July | Healthy / July | 18.43 | 0.002 | Healthy 0.142; July 0.548 |

*Dispersion did not differ between the two diseased timepoints (May vs. July F = 1.75, p = 0.20), and the corresponding PERMANOVA centroid contrast was also non-significant (p = 0.542); no dispersion-level or centroid-level change between the diseased timepoints is therefore supported.*

**Distance-metric sensitivity analysis (healthy vs. early-treatment diseased, Healthy vs. May).**

The significant healthy-vs.-diseased separation detected by ASV-level Bray–Curtis PERMANOVA was re-evaluated using compositionally aware distances (Aitchison distance on CLR-transformed counts with a pseudocount of 0.5; a robust CLR variant; and prevalence-filtered genus subsets). The separation was not reproduced under any compositional metric, and significant dispersion heterogeneity (PERMDISP) was present under both Bray–Curtis and CLR distances, indicating that the Bray–Curtis result is partly confounded by within-group dispersion and is not robust to distance-metric choice. No genus survived Benjamini–Hochberg FDR correction under CLR-based differential-abundance testing.

| **Distance metric** | **Features** | **Healthy vs. May R²** | **p** | **PERMDISP F** | **PERMDISP p** |
| --- | --- | --- | --- | --- | --- |
| Bray–Curtis (ASV-level; main text) | 238 ASVs | 0.248 | 0.001 | 127.34 | <0.001 |
| Aitchison CLR (+0.5 pseudocount) | 87 genera | 0.089 | 0.345 | 6.42 | 0.009 |
| Robust CLR (rCLR) | 87 genera | 0.083 | 0.443 | 8.55 | 0.003 |
| CLR, prevalence ≥3/23 | 36 genera | 0.049 | 0.921 | — | — |
| CLR, prevalence ≥6/23 | 18 genera | 0.053 | 0.804 | — | — |
| CLR, prevalence ≥12/23 | 5 genera | 0.063 | 0.625 | — | — |

**Supplementary Table S6. Per-library read counts and per-sample DADA2 processing statistics for the 23 ITS2 amplicon libraries.**

Read counts at each stage of the QIIME 2 (v2025.7.0) processing pipeline for the 23 ITS2 amplicon libraries deposited under BioProject PRJNA1464532. Raw reads are the counts held by NCBI for the deposited runs (mean read length 301 bp); quality-filtered counts follow quality-filter q-score; DADA2-filtered, denoised and non-chimeric counts follow dada2 denoise-single; final counts follow removal of mitochondrial, chloroplast and kingdom-level-unassigned features. Sample IDs, BioSample and Run accessions correspond to Supplementary Table S3. Group denotes the treatment group of Supplementary Table S3 (Healthy, May or July); Final denotes the reads remaining after taxonomic filtering; ASVs denotes the observed ASVs after rarefaction.

| **Sample ID** | **Group** | **Region** | **BioSample (SAMN)** | **Run (SRR)** | **Raw** | **Quality-filtered** | **DADA2-filtered** | **Denoised** | **Non-chimeric** | **Final** | **ASVs** |
| --- | --- | --- | --- | --- | --- | --- | --- | --- | --- | --- | --- |
| GW_D_May_8 | May | Gunwi | SAMN59720672 | SRR38495250 | 18,094 | 18,094 | 16,502 | 15,934 | 13,009 | 11,836 | 37 |
| GW_D_May_9 | May | Gunwi | SAMN59720673 | SRR38495249 | 20,150 | 20,150 | 11,793 | 11,641 | 9,761 | 7,868 | 22 |
| GW_D_Jul_8 | July | Gunwi | SAMN59720674 | SRR38495238 | 18,183 | 18,183 | 16,801 | 16,523 | 15,722 | 14,707 | 21 |
| GW_D_Jul_9 | July | Gunwi | SAMN59720675 | SRR38495234 | 16,516 | 16,516 | 15,573 | 15,446 | 12,979 | 11,768 | 18 |
| GW_E_May_5 | May | Gunwi | SAMN59720676 | SRR38495233 | 19,186 | 19,186 | 17,695 | 17,252 | 15,952 | 15,302 | 27 |
| GW_F_May_3 | May | Gunwi | SAMN59720677 | SRR38495232 | 19,725 | 19,725 | 17,084 | 16,937 | 16,513 | 13,450 | 42 |
| NW_B_May_3 | May | Namwon | SAMN59720678 | SRR38495231 | 22,992 | 22,992 | 20,721 | 20,537 | 20,537 | 18,718 | 44 |
| GW_E_Jul_5 | July | Gunwi | SAMN59720679 | SRR38495230 | 24,202 | 24,202 | 22,610 | 22,195 | 22,090 | 20,942 | 24 |
| GW_F_Jul_3 | July | Gunwi | SAMN59720680 | SRR38495229 | 26,675 | 26,675 | 25,627 | 25,369 | 24,851 | 23,269 | 32 |
| NW_B_Jul_3 | July | Namwon | SAMN59720681 | SRR38495228 | 21,643 | 21,643 | 19,839 | 19,687 | 15,259 | 12,882 | 45 |
| CJ_D_May_5 | May | Chungju | SAMN59720682 | SRR38495248 | 24,280 | 24,280 | 23,401 | 23,254 | 22,469 | 21,777 | 33 |
| CJ_E_May_1 | May | Chungju | SAMN59720683 | SRR38495247 | 26,871 | 26,871 | 25,408 | 25,278 | 23,637 | 21,329 | 19 |
| CJ_D_Jul_5 | July | Chungju | SAMN59720684 | SRR38495246 | 21,518 | 21,518 | 19,944 | 19,800 | 19,698 | 18,544 | 19 |
| CJ_E_Jul_1 | July | Chungju | SAMN59720685 | SRR38495245 | 21,059 | 21,059 | 20,048 | 19,985 | 19,329 | 18,972 | 23 |
| CJ_E_Jul_4 | July | Chungju | SAMN59720686 | SRR38495244 | 15,748 | 15,748 | 14,709 | 14,641 | 14,477 | 13,805 | 22 |
| IC_A_May_2 | May | Icheon | SAMN59720687 | SRR38495243 | 18,871 | 18,871 | 15,844 | 15,745 | 15,393 | 11,728 | 31 |
| IC_C_May_3 | May | Icheon | SAMN59720688 | SRR38495242 | 17,103 | 17,103 | 16,554 | 16,366 | 15,313 | 14,837 | 15 |
| IC_A_Jul_2 | July | Icheon | SAMN59720689 | SRR38495241 | 18,059 | 18,059 | 16,904 | 16,855 | 16,171 | 15,185 | 12 |
| IC_C_Jul_3 | July | Icheon | SAMN59720690 | SRR38495240 | 20,315 | 20,315 | 17,677 | 17,423 | 17,423 | 14,886 | 32 |
| GW_A_May_ctrl | Healthy | Gunwi | SAMN59720691 | SRR38495239 | 16,786 | 16,786 | 15,811 | 15,729 | 15,664 | 15,051 | 32 |
| GW_C_May_ctrl | Healthy | Gunwi | SAMN59720692 | SRR38495237 | 15,728 | 15,728 | 14,714 | 14,669 | 14,669 | 14,013 | 10 |
| CJ_A_May_ctrl | Healthy | Chungju | SAMN59720693 | SRR38495236 | 15,449 | 15,449 | 14,430 | 14,372 | 14,372 | 13,720 | 12 |
| IC_A_May_ctrl | Healthy | Icheon | SAMN59720694 | SRR38495235 | 15,483 | 15,483 | 14,317 | 14,206 | 14,206 | 13,457 | 8 |

Across the 23 libraries, 454,636 raw reads yielded 389,494 non-chimeric reads and 358,046 reads after taxonomic filtering; per library, a mean of 91.0% of raw reads passed DADA2 quality filtering and 85.7% were retained as non-chimeric. Final library sizes ranged from 7,868 to 23,269 reads (mean 15,567), so the 7,800-read rarefaction depth retained 23 of 23 libraries, and 238 ASVs were recovered across the dataset. Of 30 bark samples submitted for sequencing, 7 returned no sequence data and were excluded; the 23 libraries listed here are the complete set obtained.
